# Prediction of Novel Bacterial Small RNAs From RIL-Seq RNA–RNA Interaction Data

**DOI:** 10.3389/fmicb.2021.635070

**Published:** 2021-05-21

**Authors:** Amir Bar, Liron Argaman, Yael Altuvia, Hanah Margalit

**Affiliations:** Department of Microbiology and Molecular Genetics, Institute for Medical Research Israel-Canada, Faculty of Medicine, The Hebrew University of Jerusalem, Jerusalem, Israel

**Keywords:** sRNA (small RNA), RIL-seq, prediction, *E. coli* – *Escherichia coli*, post-transcriptional regulation, Hfq

## Abstract

The genomic revolution and subsequent advances in large-scale genomic and transcriptomic technologies highlighted hidden genomic treasures. Among them stand out non-coding small RNAs (sRNAs), shown to play important roles in post-transcriptional regulation of gene expression in both pro- and eukaryotes. Bacterial sRNA-encoding genes were initially identified in intergenic regions, but recent evidence suggest that they can be encoded within other, well-defined, genomic elements. This notion was strongly supported by data generated by RIL-seq, a RNA-seq-based methodology we recently developed for deciphering chaperon-dependent sRNA-target networks in bacteria. Applying RIL-seq to Hfq-bound RNAs in *Escherichia coli*, we found that ∼64% of the detected RNA pairs involved known sRNAs, suggesting that yet unknown sRNAs may be included in the ∼36% remaining pairs. To determine the latter, we first tested and refined a set of quantitative features derived from RIL-seq data, which distinguish between Hfq-dependent sRNAs and “other RNAs”. We then incorporated these features in a machine learning-based algorithm that predicts novel sRNAs from RIL-seq data, and identified high-scoring candidates encoded in various genomic regions, mostly intergenic regions and 3′ untranslated regions, but also 5′ untranslated regions and coding sequences. Several candidates were further tested and verified by northern blot analysis as Hfq-dependent sRNAs. Our study reinforces the emerging concept that sRNAs are encoded within various genomic elements, and provides a computational framework for the detection of additional sRNAs in Hfq RIL-seq data of *E. coli* grown under different conditions and of other bacteria manifesting Hfq-mediated sRNA-target interactions.

## Introduction

Trans-acting small RNAs (sRNAs) have emerged as a major class of post-transcriptional gene expression regulators in bacteria. These are short RNA molecules, 50–400 nucleotides long, which regulate their targets in trans, usually by incomplete base pairing with their mRNAs, affecting translation and/or mRNA stability (Wagner and Romby, 2015; Hör et al., 2020). sRNAs were discovered in many bacteria and were shown to play regulatory roles in diverse cellular processes, and in particular in the response to various stress conditions. Often, these RNA regulators are associated with chaperon proteins, such as Hfq (Vogel and Luisi, 2011) or ProQ (Melamed et al., 2020). In many Gram-negative bacteria the protein chaperon Hfq mediates many of the sRNA-target interactions and stabilizes the sRNAs (Vogel and Luisi, 2011; De Lay et al., 2013; Updegrove et al., 2016; Santiago-Frangos and Woodson, 2018). Yet, there are sRNAs in *Escherichia coli* for which it was suggested that their RNA-binding activity is Hfq-independent (Mihailovic et al., 2018). In the present study, we focus on Hfq-dependent sRNAs in *E. coli*.

While the initial discovery of the first sRNA in *E. coli*, Spot 42, is dated to 1973 (Ikemura and Dahlberg, 1973a, b) and a few other sRNAs were discovered serendipitously along the years [e.g., MicF (Mizuno et al., 1984), DsrA (Sledjeski and Gottesman, 1995), OxyS (Altuvia et al., 1997)], their big burst occurred following the genomic revolution in the mid-1990s. The completion of the genome sequencing of *E. coli* inspired several systematic computational-experimental expeditions, attempting to identify additional sRNA-encoding genes based on the genome information. As all previously known sRNAs were encoded by genes located between two protein coding genes, the initial screens were focused at intergenic regions and identified novel sRNA-encoding genes only in those regions (Argaman et al., 2001; Rivas et al., 2001; Wassarman et al., 2001; Chen et al., 2002). Yet, subsequent experimental screens of RNAs bound to Hfq, carried out in several bacterial species, revealed putative Hfq-bound sRNAs encoded in various genomic regions, including coding sequences (CDS) and 5′ and 3′ untranslated regions (UTR) (Zhang et al., 2003; Chao et al., 2012; Bilusic et al., 2014; Tree et al., 2014; Huber et al., 2020). These sRNAs may be either independently transcribed, or processed from mRNAs by endoribonucleases (Miyakoshi et al., 2015b). When processed from the mRNA of their hosting gene they often regulate genes involved in the same pathways as the parent gene and may generate efficient regulatory circuits [e.g., CpxQ and *cpxP*, and GadF and *gadE* (Chao and Vogel, 2016; Grabowicz et al., 2016; Melamed et al., 2016)].

The discovery of novel Hfq-bound sRNAs that are encoded within a variety of genomic elements was enhanced by RIL-seq (RNA Interaction by Ligation and sequencing), a high-throughput methodology we recently developed for mapping direct RNA–RNA interactions mediated by Hfq (Melamed et al., 2016, 2018). The idea behind RIL-seq is that a sRNA and a target RNA co-bound to Hfq could be ligated and then identified by sequencing as chimeric fragments. The major steps of RIL-seq involve *in vivo* protein-RNA crosslinking, co-immunoprecipitation of Hfq and bound RNAs, RNA ligation and paired-end RNA sequencing. Interacting pairs are identified by mapping the ends of sequenced fragments to the genome and identifying chimeric fragments in which the two ends are mapped to two different genomic locations. Only chimeric fragments whose abundance exceeds random expectation are kept and considered as representing RNA interacting pairs (statistically significant chimeras, hereinafter, S-chimeras). Application of RIL-seq to *E. coli* grown to exponential phase, stationary phase and exponential phase under iron limitation revealed ∼2800 RNA–RNA interactions, ∼64% of which involved well-established sRNAs and the rest involved RNAs derived from various genomic entities (Melamed et al., 2016). Interestingly, in most of the chimeric fragments including known sRNAs, the sRNA was the second RNA in the chimera (at the 3′ part of the chimeric RNA). This regarded both class I and class II sRNAs (Schu et al., 2015). The positioning of the sRNAs as second in the chimeras is consistent with the known binding mode of many sRNAs within Hfq, where the uridine-rich 3′ terminus of the sRNA (hereinafter, U-tract) is bound by Hfq (Otaka et al., 2011; Sauer and Weichenrieder, 2011; Dimastrogiovanni et al., 2014; Schu et al., 2015). RIL-seq involves, prior to the ligation of Hfq-bound RNAs, a step where RNA regions that are not protected by Hfq or by base pairing are trimmed by riboendonucleases and treated with polynucleotide kinase, generating 5′P end of the sRNA. This 5′P end is accessible to the ligase, resulting in chimeras where the sRNA is the second RNA. In fact, this finding provided further support to the suggested binding mode of sRNAs on Hfq (Dimastrogiovanni et al., 2014), by the identification of a common motif in the second RNAs of RIL-seq chimeras, comprising a GC-rich sequence followed by a U tail (Melamed et al., 2016), compatible with a transcription terminator. In addition, Holmqvist et al. (2016) identified a similar motif in mRNA 3′ UTR sequences bound by Hfq in *Salmonella*. The observation that sRNAs are often second in their respective chimeric fragments has raised the intriguing conjecture that the second RNAs in chimeric fragments that do not contain known sRNAs may be novel sRNAs. Furthermore, many of the RNAs found at the 3′ part of the chimeric fragments (second RNAs of the chimeras) were derived from intergenic regions or from 3′ UTRs, underpinning their potential as novel sRNAs. Indeed, some of these second RNAs, such as those embedded in the 3′ UTR of *cutC* and in the 3′ UTR of *cpxP* were identified in independent studies as sRNAs (Guo et al., 2014; Chao and Vogel, 2016).

In total, RIL-seq data comprised ∼1000 RNA–RNA pairs that did not include a known sRNA (Melamed et al., 2016), suggesting that they may include yet unknown sRNAs. To identify novel sRNAs systematically, we characterized the RNAs in all RIL-seq chimeras by various features inferred from the data and from their sequences. The distributions of several of these features, such as the number of unique interactions that a RNA is involved in, were found to differ statistically significantly between known sRNAs and “other RNAs”, reaffirming them as informative features. Here, we describe and discuss the set of informative features of sRNAs as well as a predictive algorithm utilizing them, provide a list of potential novel sRNAs and report the experimental verification of novel sRNAs encoded in intergenic regions within operons, in 5′ and 3′ UTRs and within the coding sequence. Our computational and experimental results support the expanding concept that there is a reservoir of sRNAs encoded within a variety of genomic entities and expressed under various conditions (Adams and Storz, 2020; Adams et al., 2021). The computational framework that we provide for analysis of Hfq RIL-seq data can be used to identify novel sRNAs in RIL-seq data generated for *E. coli* grown under additional cellular conditions and in RIL-seq data generated for other bacteria manifesting Hfq-mediated sRNA-target interactions. It may also inspire the application of similar algorithms for analysis of large-scale data generated by equivalent protocols in other contexts.

## Materials and Methods

### Computational Analysis

#### Data

We used three data sets of chimeric fragments corresponding to S-chimeras, obtained in RIL-seq experiments applied to bacteria grown to exponential phase, to stationary phase and to exponential phase under iron limitation (Melamed et al., 2016). The data set of exponential growth phase was obtained from six biological replicates of the experiment, while the data sets of the stationary phase and growth under iron limitation were obtained from three biological replicates in each condition. Each RNA in the data was annotated as either “known sRNA” or “other RNA” (Supplementary Table 1). We included in the set of known sRNAs all RNAs that were annotated as sRNAs prior or in parallel to RIL-seq publication (Melamed et al., 2016). The latter regard CpxQ (Chao and Vogel, 2016), SroC (Miyakoshi et al., 2015a) and 3′ETS-leuZ (Lalaouna et al., 2015). Any RNA that is not a known sRNA was annotated as “other RNA”. The total numbers of known sRNAs and “other RNAs” in each group within each of the three data sets is summarized in Table 1.

**TABLE 1:** Number of sRNAs and “other RNAs” in the various data sets.

**Condition/growth phase**	**Number of known sRNAs**	**Number of “other RNAs”**
Exponential phase	26	751
Stationary phase	29	1201
Exponential phase under iron limitation	29	1248

#### Selecting Features Distinguishing sRNAs From “Other RNAs”

We describe each RNA by features mainly extracted from RIL-seq data (Supplementary Table 2) and compare their distributions between the groups of sRNAs and “other RNAs” by Mann–Whitney U test (with Bonferroni correction for multiple hypotheses testing). For features that differ statistically significantly between the two groups we compute the Pearson correlation coefficient between every pair of features, cluster the features based on their correlation coefficients, and select one of the features in a cluster as representative. As the data corresponding to a RNA in one RIL-seq experiment (e.g., exponential phase) may differ from the data corresponding to this RNA in another RIL-seq experiment (e.g., stationary phase), all analyses were carried out separately for each data set. We verified that the selected features were found to statistically significantly differ between the group of known sRNAs and the group of “other RNAs” in all data sets. These selected features were used in the successive analyses.

#### Predicting the Probability of a RNA to Be a sRNA

The development of the predictive scheme was carried out separately for each data set. Each RNA in the data was described by a vector of the selected features. The data set was split in a ratio of 2:1 into a training set and a test set, respectively, where in each set the ratio of known sRNAs to “other RNAs” was maintained (Table 1). We applied logistic regression (python sklearn module) to the training set. The logistic regression provides weights to the different features (β_*i*_) and an intercept (β_0_), such that $l=\beta_{0}+\sum_{i=1}^{n}\beta_{i}\cdot x_{i}$, where *n* is the number of selected features and *x_i* is the value of feature *i*. The probability of a RNA to be a sRNA is then computed as 1/(1 + exp(−*l*)). We tested the obtained logistic regression model by applying it to RNAs in the test set. In practice, we conducted 10,000 iterations of this procedure, and recorded the probabilities a RNA obtained when it was included in the test set of an iteration. The final predicted probability of each RNA was computed as the mean of the predicted probabilities across all the iterations in which it was included in the test set. The logistic regression was trained using the default parameters of the sklearn linear_model LogisticRegression class, i.e., using L2 regularization.

#### Computation of Feature Contribution

It is common to examine the weights in order to learn on the relative contributions of the various features to the computed probability. However, as the values of the different features span different numeric scales, comparison of the weights *per se* is not informative. Instead, we can transform the feature values into z-scores, and compare the products of weight and the feature standard deviation:

$$\beta_{0}+\sum_{i=1}^{n}\beta_{i}\cdot x_{i}=\beta_{0}+\sum_{i=1}^{n}\beta_{i}\cdot \left(x_{i}+m_{i}-m_{i}\right)=\left(\beta_{0}+\sum_{i=1}^{n}\beta_{i}\cdot m_{i}\right)+\sum_{i=1}^{n}\beta_{i}\cdot \left(x_{i}-m_{i}\right)=\left(\beta_{0}+\sum_{i=1}^{n}\beta_{i}\cdot m_{i}\right)+\sum_{i=1}^{n}\beta_{i}\cdot \frac{s_{i}}{s_{i}}\cdot \left(x_{i}-m_{i}\right)=\left(\beta_{0}+\sum_{i=1}^{n}\beta_{i}\cdot m_{i}\right)+\sum_{i=1}^{n}\beta_{i}\cdot s_{i}\cdot zscore\left(x_{i}\right)$$

where *m_i_* and *s_i_* are the mean and the standard deviation, respectively, of the RNA’s *i^th^* feature values. The equation shows that transforming the data to z-scores is associated by an appropriate change of the intercept by the weighted sum of the mean feature values, and the weights of the features are represented by the products of the original weight and standard deviation of each feature, which are comparable. In practice, we applied this transformation to the average coefficients from the 10,000 logistic regression iterations we conducted per growth condition.

#### Principal Component Analysis (PCA)

Feature vectors of the RNAs were initially scaled with python’s sklearn preprocessing module using the *robust_scale* function. Then, we applied the PCA transformation for the first two dimensions using sklearn decomposition PCA class.

#### 5′ and 3′ Boundaries of sRNA Transcripts

5′ and 3′ transcript boundaries of the recently published sRNAs relied on the original papers reporting them (Table 2A). 5′ and 3′ transcript boundaries of the new sRNAs predicted here were determined based on the read coverage in corresponding RNA-seq libraries (see below). For *fadA* 3′ UTR we were not able to determine the 5′ end (marked unknown in Table 2B) and estimated its 5′ end position based on the size of the band observed in the Hfq-dependent northern blot (see below).

**TABLE 2:** Novel sRNAs.

**A. Novel sRNAs predicted based on RIL-seq results and recently reported in published papers**								
**Novel**	**Hosting gene**	**Genomic**	**Genomic**	**Number of**	**Prediction score**			**Comment/**
**sRNA**	**or operon**	**region**	**position**	**unique targets**	**(probability of being a sRNA)^a^**			**References**
					**Exponential**	**Stationary**	**Iron limitation**	
FlgO	*flgL*	3′ UTR	1,140,986 → 1,141,063	4	0.073	0.087	0.152	Hör et al. (2020)^b^
FliX	*FliC*	3′ UTR	2,001,912 ← 2,002,106	17	0.859	0.272	0.672	Hör et al. (2020)^b^
GadF	*gadE*	3′ UTR	3,658,992 → 3,659,082	24	–	0.684	–	Melamed et al. (2016)
MalH	*malG*	3′ UTR	4,242,531 ← 4,242,629 or 4,242,531 ← 4,242,633^*c*^	38	0.416	0.468	0.596	Iosub et al. (2020)
MotR	*motA*	5′ UTR	1,977,208 ← 1,977,300	19	0.562	0.158	0.444	Hör et al. (2020)
NarS-L	*narK*	3′ UTR	1,279,286 → 1,279,520	8	0.376	–	0.380	Wang et al. (2020)
NarS-S			1,279,337 → 1,279,520					
PspH	*pspG*	3′ UTR	4,263,139 → 4,263,249	1	0.003	0.002	0.001	Melamed et al. (2016)
RaiZ	*raiA*	3′ UTR	2,737,381 → 2,737,542	29	0.337	0.482	0.296	Smirnov et al. (2017)
RaiZ-S			2,737,417 → 2,737,542					
RbsZ	*rbsB*-*rbsK*	Intergenic in operon	3,937,045 → 3,937,278	4	0.091	0.035	0.105	Melamed et al. (2020)
SdhX (RybD)	*sucD*	3′ UTR	765,050 → 765,150	33	0.638	0.836	0.735	De Mets et al. (2019); Miyakoshi et al. (2019)
UhpU	*uhpT*	3′ UTR	3,845,730 ← 3,845,995	111	0.751	0.347	0.786	Hör et al. (2020)^*b*^

**B. Novel sRNA candidates predicted based on RIL-seq results and verified by northern blot analysis in the current study**								

AceK-int	*aceK*	CDS	4,218,879 → 4,218,963	15	–	0.592	0.084	Recently verified also by Adams et al. (2021)
AllZ	*allR*	3′ UTR	533,629 → 533,863	5	0.042	0.295	0.046	
BhsB	*bhs*A	3′ UTR	1,169,303 → 1,169,402	2	–	0.099	–	
FadZ	*fadA*	3′ UTR	4,027,232 ← unknown	12	0.031	0.202	0.081	
KilS	*kilR*	5′ UTR	1,418,405 ← 1,418,502	3	0.033	0.154	0.032	
XylZ	*xylA-xylB*	Intergenic in operon	3,729,386 ← 3,729,545	10	0.064	0.346	0.068	
ZbiJ	*ybiJ*	3′ UTR	837,435 ← 837,531	21	0.223	0.374	0.506	Recently verified also by Han and Lory (2021), who called it “asYbiE”

*^a^Dashed cells mean the RNA was not included in the data of the corresponding experiment.*

*^b^Referring to unpublished results from Storz’s lab.*

*^c^Mapping of the 5′ end is according to Iosub et al. (2020). According to our RNA-seq results the 5′ end is at 4,242,609.*

#### Identification of Transcription Start Sites and RNase E Cleavage Sites Near Predicted sRNAs

In order to appreciate if the transcripts of the novel sRNAs were generated by independent transcription or by cleavage of the hosting mRNA, we used published data of large-scale screens of transcription start sites (TSSs) (Thomason et al., 2015; Ju et al., 2019) and RNase E cleavage sites (Clarke et al., 2014), and searched for TSSs and cleavage sites located between the determined 3′ end and up to 50 nucleotides upstream the 5′ end.

#### Identification of Putative sRNAs in Hfq-CLASH Data

To verify whether the putative sRNAs we report are supported by other data sets, we compared their estimated coordinates to chimeric fragments included in the Hfq-CLASH data set (Iosub et al., 2020). We considered a RNA as found in Hfq-CLASH chimera if its estimated coordinates were at most 50-nt apart from the coordinates reported in Iosub et al. (2020).

### Experimental Testing

#### Strains and Growth Conditions

For the verification of novel sRNA expression, cultures of *Escherichia coli* MG1655 and its isogenic strain MG1655 *hfq::Kn* were grown over-night in LB medium and then diluted 1:100 in fresh LB medium and grown while shaking at 37°C. Samples of culture were collected throughout growth, and centrifuged at 4°C. The pelleted cells were resuspended in 50 μl of TE buffer (10 mM Tris HCl pH 8.0, 1 mM EDTA pH 8.0), mixed with lysozyme to a final concentration of 0.9 mg/ml and fast frozen in liquid nitrogen. The samples were then subjected to two cycles of thawing at 37°C and freezing in liquid nitrogen.

#### Northern Analysis

Total RNA was extracted from harvested cells using TRI-reagent (Sigma). 30 μg of total RNA were separated in 7 M urea/6% polyacrylamide gels in 44.5 mM Tris-base, 44.5 mM boric acid and 2 mM EDTA pH 8.0, and transferred to Zeta-Probe membrane (Bio-Rad) by electroblotting. The membranes were hybridized with specific [^32^P] end labeled DNA probes. For each tested sRNA, the northern blot was repeated at least twice, with a different replicate of total RNA. The probe sequences are listed in Supplementary Table 3.

#### RNA-Seq

We used compatible RNA-seq data available in the lab, which were generated as following: Three single colonies of MG1655 cells carrying a pJV300 plasmid (Urban and Vogel, 2007) were grown over night at 37°C in LB medium supplied with Ampicillin (100 μg/ml). The cultures were diluted 1:100 in fresh medium and grown while shaking at 37°C for 6 h. Cells were collected and RNA was extracted as described above. RNA-seq libraries were constructed according to the RNAtag-seq protocol (Shishkin et al., 2015), with few modifications described in Melamed et al. (2018). The libraries were paired-end sequenced using Illumina NextSeq 500 machine, with read length of 45 and 40 bp for first and second read, respectively. Raw reads were split into their original three replicate libraries using an in-house script. Cutadpat was applied to remove adapter sequences, low quality ends and sequences shorter than 25 nucleotides (Martin, 2011). We applied bwa aln followed by bwa sampe (Li, 2013) to align the reads to the genome. We applied stringent mapping allowing only two mismatches. The total number of reads in the libraries of the three replicates 1, 2, 3 was 10674656, 16274638, 10368982 reads, respectively. In all three libraries 99% of the reads passed the processing filter and 89% of the processed reads were successfully mapped. Library 2, which had the highest number of reads, was used to define the novel sRNA boundaries.

## Results

Examination of the chimeric fragments corresponding to S-chimeras in RIL-seq data hinted at several properties that may aid in the classification of RNAs represented in these chimeras as either sRNA or target RNA (Melamed et al., 2016). sRNAs were included in many chimeric fragments, were found to interact with multiple targets and were preferentially identified as the second RNA in the chimeric fragments. In contrast, RNAs found in interaction with a known sRNA were usually found to be involved in a small number of chimeric fragments, were found to interact with only a few partners (mainly, the sRNA), and were frequently identified as the first RNA in the chimeric fragments.

While previously these properties were intuitively considered for supporting or rejecting a sRNA candidate (Melamed et al., 2016), our aim here is to quantify them and carry out a systematic analysis, selecting informative features that will be incorporated in a sRNA predictive scheme. The features that we propose to examine for each RNA are of two types: (*i*) features derived from the chimeric fragments the RNA is involved in (first layer), and (*ii*) features of the RNA interactors (second layer). The incorporation of both layers of features in the analysis is inspired by the acknowledgment that up to date the number of identified sRNA–sRNA interactions is very small and far below the number of identified sRNA interactions with mRNAs. Recognizing first layer features that support the RNA as a sRNA along with second layer features that do not support the interactors as sRNAs should provide stronger support for a sRNA candidate than expected from its first layer features alone. Combining the two layers of traits is expected to enhance the discriminative power of the model and to increase the reliability of predicted sRNAs.

### Feature Selection

All analyses described hereinafter were carried out for each RIL-seq data set separately (exponential phase, stationary phase, exponential phase under iron limitation). We describe in the text the results for the stationary phase data and in the Supplementary Material the results for the two other data sets. When relating to the chimeric fragments, we refer to chimeric fragments corresponding to S-chimeras identified in RIL-seq results.

Some of the traits characterizing a RNA can be quantitatively described in several ways (hereinafter, features). For example, let X be a RNA that was identified as interacting with k RNAs *Y*_1_,…,*Y*_*i*_,…,*Y*_*k*_ and is involved in *n*_1_,…,*n*_*i*_,…,*n*_*k*_ chimeric fragments corresponding to each RNA, respectively, making up a total of N chimeric fragments. The trait ‘number of chimeric fragments the RNA is involved in’ can be described as N, or as the mean of n_*i*_, or as the median of n_*i*_. We examined 18 features in total, several of which regard different representations of the same trait (Supplementary Table 2). We assigned each RNA the values of the features. For each feature we compared the distributions of its values between the group of known sRNAs and “other RNAs” by two-tailed Mann–Whitney U test with Bonferroni correction for multiple hypotheses testing (Supplementary Material and Supplementary Table 2). We then clustered all features that differed statistically significantly between the group of known sRNAs and group of “other RNAs” (Supplementary Material and Supplementary Figure 1), and selected from each cluster of features one representative feature (usually the one with simplest intuitive interpretation) to be used in successive analyses. In addition to features solely based on RIL-seq data, we also included the length of the U-tract of the RNA, as we previously observed that sRNAs have longer U-tracts at their terminators compared to “other RNAs” (Melamed et al., 2016). The U-tract length is also considered a first-layer feature, as it is a feature of the RNA itself.

**FIGURE 1:**
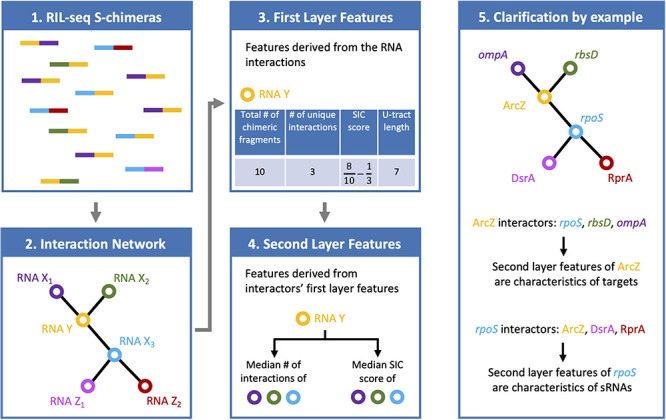
Features characterizing a RNA in RIL-seq data. RIL-seq final data set includes chimeric fragments corresponding to S-chimeras (first box), determining RNA interacting pairs, which can be described as a RNA–RNA interaction network (second box). Each node in the network represents a RNA. Two nodes are connected by an edge if they were determined as interacting. For every RNA, first layer features were computed based on the chimeric fragments it was involved in (third box; U-tract length is not shown on the chimeric fragments). Finally, for each RNA, second layer features were computed based on the first layer features of its interacting partners (forth box). If the RNA is a sRNA, its first layer features are expected to be characteristic of a sRNA, namely, many chimeric fragments, many interactions, high fractions of chimeras in which it is second RNA (high SIC, Second-In-Chimera, score), long U-tract. The second layer features of the sRNA, which involve the first-layer features of its interactors, are expected to be characteristic of targets, namely, low number of interactions and low SIC scores (fifth box).

Six features were selected, four are first layer features and two are second layer features (Figure 1). It is of note that these six features were consistently selected in the analyses of all three data sets. For each RNA these features are: (A) *Total number of chimeric fragments*: The total number of chimeric fragments that included the RNA. This value was normalized by the total number of chimeric fragments in the data set. (B) *Number of unique interactions*: Number of unique interactions the RNA was involved in (k). This value was normalized by the total number of unique interactions in the data set. (C) *Second-In-Chimera (SIC) score*: A score representing the fraction of chimeric fragments in which the RNA was the second RNA of the chimera, while taking into account the number of unique interactions this RNA is involved in. We defined this score as $S-\frac{1}{k}$, where S is the fraction of chimeric fragments in which the RNA was the second RNA of the chimera and k is as defined above. Intuitively, for RNAs with many interactions the score is approximately S, while the score of RNAs with a small number of interactions is penalized to prevent high SIC scores that are based on one or only a few interactions. Note that due to this correction SIC may also get negative values. (D) *U-tract length*: For each RNA we assigned the length of the longest U-tract that could be identified in a region spanning 50 nucleotides around the segments of this RNA in the chimeric fragments of the RIL-seq data. (E) *Median number of interactions of interactors*: Each interactor of a RNA is also annotated by feature B. We take the median of these values across all interactors of the RNA. (F) *Median SIC score of interactors*: Each interactor has a SIC score, as defined above. We take the median of these values across all interactors of the RNA. Note that since the variances of features A, B, and E were extremely large, their values were transformed to Log_10_ scale for further analysis.

**FIGURE 2:**
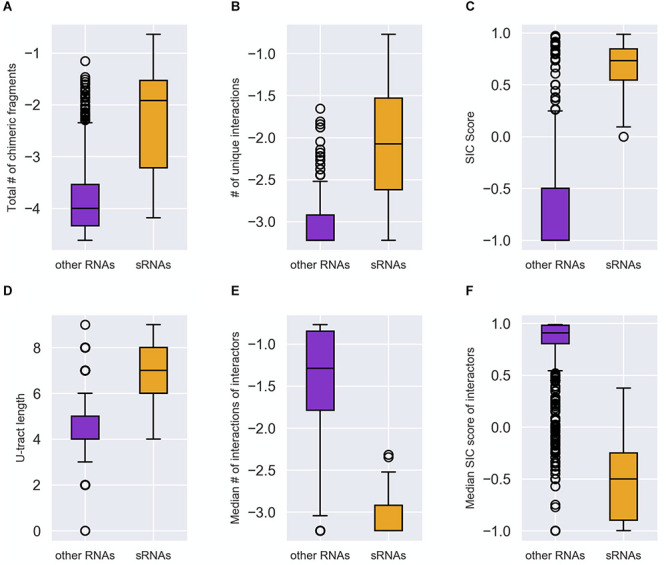
Distinction between sRNAs and “other RNAs” by the characteristic features. Each RNA was characterized by the following features: **(A)** Total number of the chimeric fragments the RNA is involved in. This value was normalized by the total number of chimeric fragments in the data set and then transformed to Log_10_ scale. **(B)** Number of unique interactions the RNA is involved in. This value was normalized by the total number of interactions in the data set and then transformed to Log_10_ scale. **(C)** SIC score (SIC, for Second-In-Chimera), namely the percentage of chimeric fragments in which the RNA was second in the chimera, penalized by the number of unique interactions the RNA is involved in. **(D)** The U-tract length of the RNA. **(E)** The median number of interacting partners of the RNA interactors (normalized as in B and expressed by Log_10_). **(F)** The median SIC score of the RNA interactors. The distributions of each feature values in the group of known sRNAs (orange) and “other RNAs” (purple) are described by boxplots. The differences between the two distributions **(A–F)** were found to be statistically significant by two-tailed Mann–Whitney U test (*p* values between 10^– 23^ and 10^– 11^ after Bonferroni correction for multiple hypotheses testing). The distributions in panels **(A–F)** are based on the data of stationary phase RIL-seq experiment (Supplementary Table 1). Results for the exponential phase data sets are shown in Supplementary Figures 2, 3.

The distributions of these feature values differed statistically significantly (after Bonferroni correction for multiple hypotheses testing) between the groups of known sRNAs and “other RNAs” (Figure 2 and Supplementary Figures 2, 3). It is evident from Figure 2 that compared to the “other RNAs”, the known sRNAs were found to be involved in more chimeric fragments and in more unique interactions; the fraction of the chimeric fragments in which they appear as second RNA is higher; and they have longer U-tracts. As for the interactors of sRNAs, the fraction of interactions in which they are second RNA in the chimera and the number of unique interactions they are involved in are lower compared to interactors of “other RNAs”. The statistically significant differences between the distributions of the features in the two RNA groups (p values between 10^– 23^ and 10^– 11^) suggested that they can be used for classifying sRNAs and for the determination of novel, yet unknown sRNAs, which may be hidden in RIL-seq data.

**FIGURE 3:**
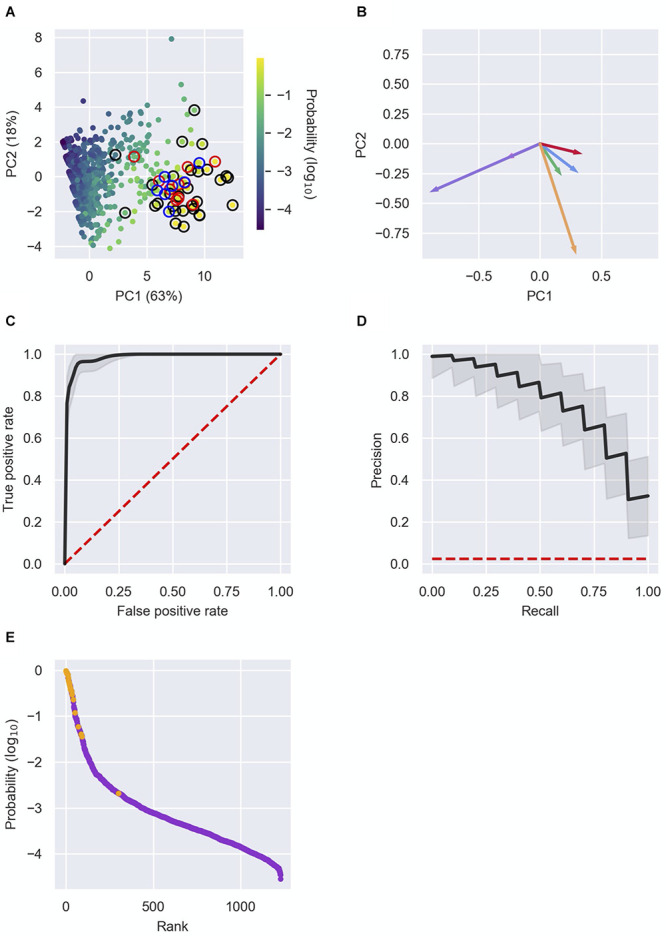
Detection of novel sRNAs. **(A)** Principal component analysis (PCA) of RNAs characterized by the six features. The RNAs (dots) are plotted in two dimensions, using their projections onto the first two principal components. Each RNA in the data is colored by its sRNA probability, as assigned by the logistic regression analysis. Colored circles surrounding the dots represent: a well-established sRNA marked in Supplementary Table 1 by 1 (black), a recently discovered sRNA listed in Table 2A (red) or a newly discovered sRNA listed in Table 2B (blue). **(B)** Contribution of the features to PC1 and PC2. The vectors represent the coefficients of the features in each PC: Total number of chimeric fragments (green), number of unique interactions (blue), SIC score (red), U-tract length (orange), median number of interactions of interactors (pink), median SIC score of interactors (purple). **(C,D)** Receiver operating characteristic (ROC) curve **(C)** and precision–recall (PR) curve **(D)** showing the high predictive power of the logistic regression model. Shown in black are the curves obtained from the mean probabilities of 10,000 iterations of the logistic regression, and the curves of individual iterations in the range of one standard deviation around the curve of mean probabilities. The curves are compared to the expected curve of a random classifier (red dashed line). The area under curve (AUC) of the ROC curve is 0.98 ± 0.01. **(E)** Known sRNAs and “other RNAs” (colored orange and purple, respectively) were ranked by their computed sRNA scores. Highly ranked RNAs, yet unknown as sRNAs, are predicted as putative novel sRNAs. Presented results are for the data set of stationary phase RIL-seq experiment. Results for the exponential phase data sets are shown in Supplementary Figures 4, 5.

Each RNA in RIL-seq data was represented by a vector of the above six features. Analysis of the vectors by principal component analysis (PCA) further demonstrated the separation of known sRNAs from “other RNAs” by the features and the contribution of the various features to this separation (Figures 3A,B and Supplementary Figures 4A,B, 5A,B). Furthermore, this analysis showed additional RNAs in close proximity to the previously known sRNAs, suggesting these might be novel sRNA candidates. Intriguingly, several novel sRNAs derived from 3′ UTRs, which were recently verified experimentally (Table 2A), are clustered together with known sRNAs in the PCA plots.

### Prediction of Novel sRNAs

To systematically and comprehensively identify novel sRNA candidates, we applied logistic regression, using these six features as characteristics of each RNA in the data. We applied 10,000 iterations of the logistic regression, where in each iteration we randomly split the data into a training set and a test set. Each training set included 2/3 of the known sRNAs and 2/3 of the “other RNAs” and the test set included the rest of the data. At each iteration we trained a logistic regression model on the training set, resulting in a linear combination of the features, which provides the probability of a RNA in the data to be a sRNA (see the section “Materials and Methods”). The model was then used to compute these probabilities for RNAs in the test set. At the end of the process, each RNA had a list of N probabilities, where N is the number of test sets that included this RNA. The final sRNA probability of a specific RNA was the average of these probabilities, considered hereinafter as the sRNA score of the RNA. Determining different probability thresholds above which a RNA is determined as a sRNA, we obtained a receiver operating characteristic (ROC) curve and a precision–recall (PR) curve for each iteration and for the average results (Figures 3C,D and Supplementary Figures 4C,D, 5C,D), showing the consistency and high predictive power provided by the logistic regression.

Due to the low frequency of sRNAs in the data and the inclusion of sRNAs not yet discovered in the training set, we expect the model to output uncalibrated prediction probabilities. We therefore did not determine a probability threshold above which a RNA is predicted as a sRNA, but ranked the RNAs by their sRNA scores, scanned the ranked RNAs from top down and searched for RNAs ranked above or in the vicinity of known sRNAs (Figure 3E and Supplementary Figures 4E, 5E). As the logistic regression was performed for each RIL-seq data set separately, the ranking of a specific RNA can change between conditions. This stems from the fact that both the feature vectors of RNAs and the annotated sRNAs that are included in a data set are condition specific. This implies a RNA can be predicted as a sRNA under one condition, but not necessarily under another condition, consistent with the acknowledged condition-specific expression of sRNAs (Wagner and Romby, 2015). In fact, we find an association between the change in expression levels of the sRNAs between conditions and the differences in their sRNA score between the corresponding conditions (Supplementary Figure 6). This implies a relationship between the expression level of the sRNA and its sRNA score per condition. For example, SroC, known to be expressed in stationary phase (Miyakoshi et al., 2015a), got a sRNA score of 0.5 in stationary phase data, but scores of 0.004 and 0.003 in the data sets of exponential phase and exponential phase under iron limitation, respectively. Encouragingly, many of the known sRNAs have obtained high ranking scores in at least one data set (Supplementary Table 1). Thus, the implementation of the selected features of the RNAs in a machine learning approach, such as the logistic regression, enables the distinction of sRNAs from “other RNAs”, and therefore may enable the discovery of novel sRNAs. Notably, we identified most of the recently discovered sRNAs (that were not annotated as such in our data) among the top ranking RNAs (Table 2A), as well as additional novel sRNAs, some of which (Table 2B) we tested experimentally, as detailed below.

To verify that our results do not depend on the number of iterations or the selected ratio of 2:1 between the sizes of the training and test sets, we conducted the analyses for different numbers of iterations and different ratios of training to test set sizes. These analyses confirmed that the results are independent of these parameters (Supplementary Material and Supplementary Figures 7–9).

### Contribution of Individual Features to the Classification

**FIGURE 4:**
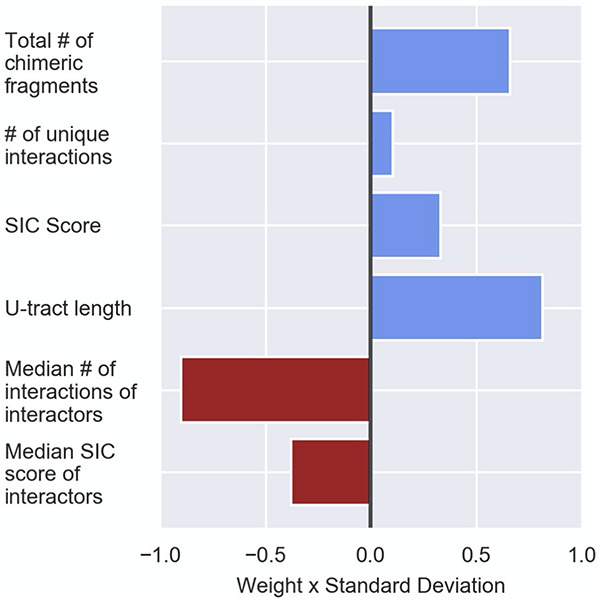
Contribution of the various features to the logistic regression predictions. Presented are the logistic regression weights after z-score transformation of the feature values (see the section “Materials and Methods”). The presented weights, which are the original weights (Table 3) multiplied by the standard deviation of the feature value, are comparable. The weight value represents its contribution to the probability the logistic regression model provides, and the sign signifies the direction in which the weight affects this probability (i.e., positive values increase the sRNA probability and negative values reduce the sRNA probability). The results are based on the data set of stationary phase RIL-seq experiment. Results for the exponential phase data sets are shown in Supplementary Figure 10.

The logistic regression assigns weights to the features, which are used for the computation of the probability of a RNA in the data to be a sRNA (see the section “Materials and Methods,” Table 3, and Supplementary Table 4). As shown in Table 3 and Supplementary Table 4, the various features differ in their contributions to the predicted probability. First, since the first-layer features directly assess a RNA as a sRNA and the second-layer features are expected to contribute to the prediction by rejecting its interactors as sRNAs (Figure 1), it is affirmative that the weights reflecting the contributions of the second layer features are in opposite signs to the contributions of the first layer features (Table 3, Figure 4, Supplementary Table 4, and Supplementary Figure 10). Secondly, as explained in the Section “Materials and Methods,” we can assess the relative contributions of the various features to the computed probability by examining the products of the weight and standard deviation of the feature values (Figure 4 and Supplementary Figure 10). It seems that the major contributors to the final sRNA score involve both first- and second-layer features. The features that are high contributors in all data sets are ‘the total number of chimeric fragments’ and the ‘median number of interactions the interactors are involved in’, while the feature that consistently has the least contribution is ‘number of unique interactions’. The contributions of the SIC (Second-In-Chimera), median SIC of interactors and the U-tract length seem to be more data set-dependent.

**TABLE 3:** Weights of the logistic regression model for stationary phase data^*a*^.

**Total number of**	**Number of**	**SIC (Second-In-Chimera)**	**U-tract**	**Median number of**	**Median SIC score**	**Intercept**
**chimeric fragments**	**unique interactions**	**score**	**length**	**interactions of interactors**	**of interactors**	
0.956	0.354	0.683	0.584	−1.345	−0.955	−5.837

*^*a*^The table shows the mean intercept and weights of the 10,000 logistic regression iterations.*

The second layer features were expected to prevent misclassification of a RNA targeted by multiple sRNAs (“target hub”) as a sRNA. To assess this, we examined the sRNA scores of “target hubs”, defined as RNAs interacting with at least four different sRNAs in at least one condition. Indeed, out of 18 “target hubs”, 16 got low sRNA scores (Supplementary Table 5). When “target hubs” present high values of first layer features, such as a long U-tract, the second layer features may not be sufficient to prevent their misclassification. Indeed, the two “target hubs” *lpp* and *ompF* have a long U-tract of eight nucleotides each, are involved in 9 and 15 unique interactions, respectively, and have many chimeric fragments, together enforcing their seemingly misclassification as a sRNAs, although with relatively low sRNA scores (Supplementary Table 1). Interestingly, a recent study identified a premature transcription termination site downstream to the transcription start site of *ompF*, suggesting that, in addition to being targeted by sRNAs in its 5′ UTR, a yet unknown small RNA overlapping *ompF* 5′ UTR might be generated (Adams et al., 2021). In general, sRNAs that function mainly as sponges of other sRNAs are not expected to be predicted by our algorithm as they usually have very few interactions (Supplementary Table 5). Yet, in a few cases the combination of various features in the prediction has allowed their identification by the computational scheme. For example, we found in RIL-seq stationary phase data that 70% of the chimeric fragments including the sRNA GcvB involve SroC, a recently discovered sRNA encoded in the 3′ UTR of *gltI*, a target of GcvB (Miyakoshi et al., 2015a). SroC sponges GcvB under stationary phase, relieving the repression of its targets. While SroC is involved mainly in the interaction with GcvB, our computational scheme awards it a relatively high sRNA probability in the stationary phase data, which is obtained by the combined contributions of all features (Supplementary Tables 1, 5).

### Experimental Verification of sRNA Candidates

**FIGURE 5:**
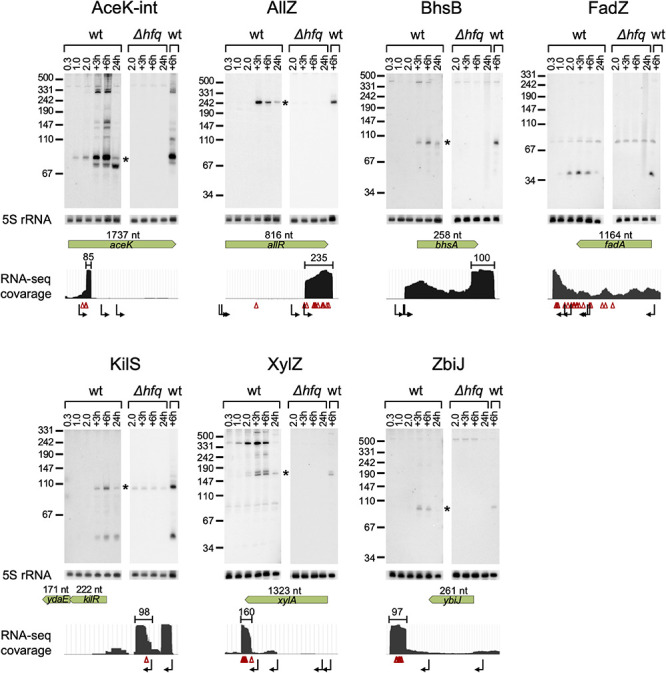
Verification of the novel sRNAs by northern analysis. Total RNA was extracted from wt *E. coli* and Δ*hfq* cultures throughout growth. Samples of the wt culture were taken at an OD_600_ of 0.3, 1.0, and 2.0, 3 h and 6 h after the culture reached an OD_600_ of 2.0 (+3 h and +6 h, respectively) and after 24 h of growth (24 h). Samples of the Δ*hfq* were taken at an OD_600_ of 2.0, 3 h and 6 h after the culture reached an OD_600_ of 2.0 and after 24 h of growth. 30 μg total RNA were subjected to northern analysis using specific probes. The membrane used for the probing of AceK-int was re-used for the probing of AllZ, after the AceK-int probe radio-labeling has faded. 5S rRNA was probed as a loading control. For each sRNA, a coverage plot of RNA-seq library made of total RNA from a stationary phase (6 h growth) culture is shown. The green arrows indicate the coding sequence (CDS) region and gene orientation, with the CDS size above the arrow in nucleotides (nt). The approximated size of each sRNA is indicated above the read coverage plot (nt). Starlet indicates the band fitting in size to the RNA-seq data. Transcription start sites, based on data of Thomason et al. (2015) and Ju et al. (2019), and RNase E cleavage sites, based on data of Clarke et al. (2014) are shown below the read coverage plots along the transcript by bent black arrows and red triangles, respectively. Transcription start and cleavage sites in the vicinity of the suspected sRNA are recorded also in Supplementary Table 6.

Our computational scheme reported newly predicted sRNAs, encoded within various genomic elements (Supplementary Table 1 summary tab). Many are encoded in 3′ UTRs, but there were also sRNA candidates encoded in 5′ UTRs and in coding sequences. We tested experimentally eleven candidates that got relatively high sRNA scores, but not necessarily those that ranked the highest above known sRNAs. These were selected to span the whole range of sRNA scores above known sRNAs and included seven candidates encoded in the 3′ UTR of protein-coding genes (*allR*, *bhsA*, *fadA*, *glpX*, *malG*, *ybiJ* and *ykgH*), two in intergenic regions (*rbsB*-*rbsK, xylA-xylB*), one in 5′ UTR (*kilR*), and one in a coding sequence (*aceK*). To validate the expression of these sRNAs we probed them by northern blotting, which provides information on both the expression pattern of the RNA and on its approximate size. Total RNA was extracted from wild type K-12 and Δ*hfq* strains grown to different growth phases and the expression of the sRNA candidates was tested. As *malG* 3′ UTR and *rbsB*-*rbsK* IGR were in the meanwhile reported by other groups as sRNAs [MalH (Iosub et al., 2020) and RbsZ (Melamed et al., 2020), respectively], we included them in Table 2A and report their northern blot results in Supplementary Figure 11. Seven of the remaining nine putative sRNAs were verified experimentally by northern blotting, where expression was evident in wild type but not in the Δ*hfq* strain (Figure 5 and Table 2B). While this paper was under revision, the expression of AceK-int was confirmed also in another publication (Adams et al., 2021). The expression of sRNAs encoded at the 3′ UTRs of *glpX* and *ykgH* could not be verified by the northern blot experiments using two different probes for each of the candidates (Supplementary Table 3). However, the accumulation of RNA-seq reads at the 3′ UTR of *ykgH* and, with a less distinct pattern, at the 3′ UTR of *glpX* hint that transcripts originating from these loci do exist independently of the hosting gene (Supplementary Figure 12). All the verified sRNA candidates obtained high sRNA scores in the analysis of RIL-seq stationary phase data, and indeed they all accumulated during the stationary growth phase (Figure 5 and Table 2B). Using RNA-seq data of stationary phase cells studied in our laboratory, we obtained estimates of the sizes of most sRNA candidates, and these sizes were confirmed by the northern blots (Figure 5).

To get clues whether the novel sRNAs were transcribed independently from an internal promoter or were processed from the hosting mRNA by an endoribonuclease, we examined global TSS data (Thomason et al., 2015; Ju et al., 2019) and large-scale cleavage data of RNase E (Clarke et al., 2014). These analyses indicated that AllZ and KilS can be transcribed from independent promoters, while the other novel sRNAs seem to be processed by an endoribonuclease from longer transcripts. We identified cleavage sites at the 5′ end position of AceK-int and two nucleotides upstream to the approximated 5′ end of XylZ (Figure 5 and Supplementary Table 6). The generation of ZbiJ and BhsB cannot be explained by the previously mapped TSSs or RNase E cleavage sites, as none were mapped near their approximated 5′ ends.

## Discussion

Systematic detection of sRNAs in large-scale RNA-seq data is highly valuable. As the fraction of genomic elements producing sRNAs out of all genes expressed in a cell is very small and estimated to be around 2% [∼100 sRNAs out of ∼4500 transcribed genes (Keseler et al., 2017)], the probability of detecting a genomic region encoding a sRNA at random is very small. In contrast, a prediction of “not a sRNA” for a genomic element has a high chance to be correct. Therefore, if our interest was in classification *per se*, it would be worthwhile to declare each RNA as non-sRNA, promising high chance of success. However, our challenge has been to find these needles in the haystack of all genes, and indeed we demonstrated that using informative features extracted from RIL-seq data and from the RNA sequences, it is feasible to distinguish the sRNAs from other genes. Using these features and the predictive scheme they are incorporated in, we predict additional novel sRNAs and demonstrate experimentally their expression as Hfq-dependent sRNAs.

**FIGURE 6:**
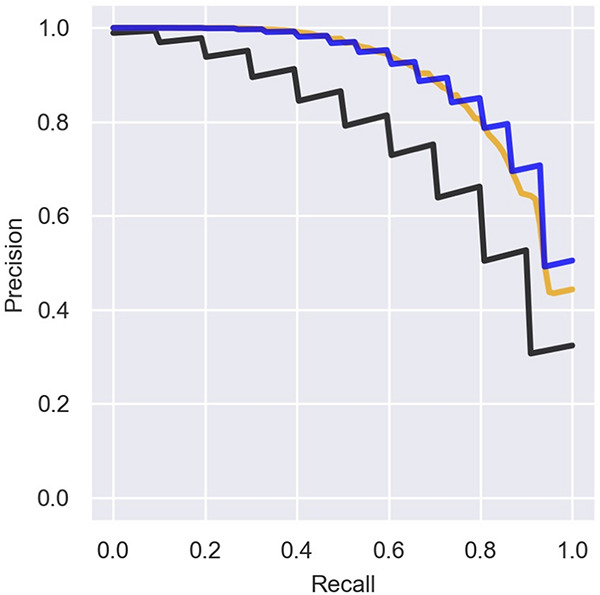
Precision of the predictions with and without labeling of novel sRNAs. Presented is a comparison of the mean precision–recall (PR) curve over the logistic regression iterations for different sets of known sRNAs. We consider three cases: (1) Precision curve as in Figure 3 (black line), when only sRNAs marked by 1 in Supplementary Table 1 are considered as known sRNAs in both training and test sets. (2) The training is done with known sRNAs as in (1) but for the assessment we label all the sRNAs in Table 2 as known sRNAs (orange line). (3) RNAs from Table 2 are labeled as sRNAs for the training and for the assessment (blue line).

There is an inherent difficulty in analyzing data that include ambiguous annotations for some genes, where some genomic elements classified as “other RNAs” are actually sRNAs that have not yet been detected. This causes the precision of the prediction to be underestimated. Indeed, if we re-label the recently discovered sRNAs and the seven additional experimentally verified sRNAs in the data (Table 2) as sRNAs and re-compute the precision rates we obtain better results (Figure 6). Interestingly, training the logistic regression model on the re-labeled data does not provide substantial improvement in the precision–recall results (Figure 6). The ambiguity of the initial labeling has also guided our strategy for determining new putative sRNAs. Thus, we chose to scan the RNAs ranked by their sRNA scores from top down, and classify RNAs ranked above known sRNAs as putative sRNAs that were wrongly labeled as “other RNAs.” Using this strategy, we predicted nine novel sRNAs that obtained sRNA scores of 0.1–0.59, seven of which were verified experimentally. As stated above, as the chance probability for a genomic element to encode a sRNA is about 0.02, a sRNA score of 0.1 is also high above random expectation.

It is interesting, yet not surprising, that a RNA can be ranked differently in the different data sets, since it gets different sRNA scores depending on the feature values and weights in each data set. As most of the feature values are derived from RIL-seq data, which may change for a particular gene from experiment to experiment, it is conceivable that its computed sRNA score may change (Supplementary Figure 6). For example, a sRNA that is weakly expressed under one of the conditions may be involved in fewer chimeric fragments under this condition, and the weak contribution of the feature “total number of chimeric fragments” may lead to a final low probability by the predictor. In no way this means that the RNA is a sRNA under one condition and not under another. It simply means that the data of this RNA under a certain condition was not sufficient to allow its identification as a sRNA. Hence, we consider a genomic element as encoding a putative sRNA if it was ranked high and among known sRNAs in at least one data set.

The computed weights are also data set-dependent, and we examined whether their relative contributions are consistent or differ among the data sets. Comparing the original weights (Supplementary Table 4) and the products of weight and standard deviation (Figure 4 and Supplementary Figure 10), we observed that, as expected, the directions of the contributions of the various features are consistent in all data sets, as well as the features that are main contributors. In all data sets the total number of chimeric fragments had a substantial positive effect, while the median number of the interactions of interactors had a large negative effect. The large contribution of this latter feature emphasizes that the recognition of the interactors as targets rather than sRNAs is highly important for the success of the predictions. Interestingly, the contribution of the U-tract length changes between the various conditions. This might be due to differences in the compositions of chimeric fragments among the various data sets, which may result in sRNAs with short U-tract in a particular data set, affecting its weight. The slight differences in the weights among the data sets suggest that it will be preferable to develop a predictive scheme per data set by repeating the learning process. Yet, the features we present can be easily extracted from the RIL-seq data and the execution of the logistic regression is straightforward, making our approach feasible for detecting novel sRNAs in *E. coli* grown under other conditions and in other bacteria to which RIL-seq is applied. It is of note that applying similar computational approaches to data sets of sRNA-target pairs detected by different methods may result in different informative features. For example, in a comparable data set of chimeric fragments including sRNAs and targets, recently determined by the CLASH methodology applied to Hfq in *E. coli* (Iosub et al., 2020), the sRNAs were not found to be preferentially the second RNAs in their respective chimeras. Encouragingly, all the novel sRNAs reported here were included in the CLASH chimeras, and half of them were located mostly second in their chimeras.

While most of the previously known sRNAs are properly classified, we do encounter and expect misclassifications emerging mainly from three major RNA classes. The first class comprises “target hubs” that interact with multiple sRNAs (Supplementary Table 5). While in most cases the second-layer features prevent their misclassification as sRNAs, some RNAs exhibiting very strong first layer features might be misclassified. Note however that the exact classification of sRNAs and targets is not always obvious and some of these allegedly misclassified sRNAs may turn out to be true sRNAs (e.g., the above described *ompF* 5′ UTR). The second class comprises sRNAs with very few targets. This group includes sRNAs that are not highly expressed in the conditions studied here and, thus, are lowly ranked at these conditions, but they are likely to be detected under the relevant condition. In addition, this class includes highly specialized sRNAs with specific targets, mostly considered as sponges. While some sponges, with extremely high first-layer properties, such as SroC, are classified as sRNAs, others are not (Supplementary Table 5). As this special class of sRNAs is not expected to be predicted by an algorithm like the one presented here, which is trained on information drawn mostly from the RNA interactome, loading the training set with single target sRNAs is not recommended. The third class of misclassifications can be traced back to ambiguous annotation of the RIL-seq data itself, and in particular to reads overlapping different genomic annotations (e.g., CDS and 3′ UTR of the same gene). Putative sRNA for which the reads are split between two annotations, are more likely to be missed. Furthermore, one annotation, e.g., CDS, can be misclassified as sRNA at the expense of the second annotation, e.g., the respective 3′ UTR-derived sRNA (e.g., *uhpT* and *sucD*). Re-examination of the proximity of the chimeric fragment coordinates of the CDS-derived candidates to 5′ UTR or 3′ UTR can resolve some of these misclassifications.

Finding that the targets of the newly revealed sRNAs have functions that are associated with the function of the hosting mRNA would support the functionality of the novel sRNAs as regulatory molecules. It would also suggest that the sRNA and hosting gene affect the same pathways at different regulation levels, and, in case they share targets they may generate regulatory circuits combining multiple regulation levels. However, as RIL-seq data do not provide information whether the sRNA enhances or represses the target expression, it would not be possible at this stage to draw mechanistic conclusions on such possible circuits. Yet, we found for several of the novel sRNAs that the hosting genes and their targets are involved in common pathways (Table 4). For example, BhsB is derived from the 3′ end of the *bhsA* mRNA, encoding a small outer membrane protein that is involved in various stress responses. Oxidative stress induced by hydrogen peroxide or paraquat activates *bhsA* transcription (Pomposiello et al., 2001; Zheng et al., 2001). Also, BhsA was shown to increase cell resistance to hydrogen peroxide (Zhang et al., 2007). The RIL-seq data indicate that BhsB interacts with two targets, *ytfK* and *ompC*, and both were shown to be involved in the cellular response to oxidative stress. *ytfK*, induced by paraquat (Pomposiello et al., 2001), was shown to be involved in hydrogen peroxide tolerance (Iwadate and Kato, 2017). OmpC, an outer membrane protein, was shown in *Salmonella* to facilitate and regulate the diffusion of hydrogen peroxide through the outer membrane (van der Heijden et al., 2016). Thus, RIL-seq results suggest a shared pathway for the hosting gene and the sRNA derived from its transcript, further supporting the functionality of the 3′ UTR-derived BhsB as a sRNA.

**TABLE 4:** Common pathways involving the host genes of novel sRNAs and their targets.

**Host gene**	**Host gene function**	**sRNA**	**sRNA target gene^*a*^**	**Target gene function**	**Suggested common pathway**	**References**	**Additional RIL-seq targets^*a*^**
*aceK*	Regulator of the branch point between the TCA cycle and the glyoxylate cycle	AceK-int	*gatY*	Tagatose-1,6-bisphosphate aldolase 2 subunit; galactitol metabolism	Carbohydrate metabolic process	LaPorte and Koshland (1982); Richet and Raibaud (1989); Nobelmann and Lengeler (1996)	*clpB.rrsG.*IGR; *glpQ*; *fur*; *ryjB*; *ryjB.sgcQ.*IGR; *ycaK.*3UTR; *ydgA*; *yfjJ*; *yqeG*
			*gatR*	*gat* operon repressor; galactitol metabolism			
			*malT*	*mal* operon activator; maltose catabolism and transport			
*allR*	Transcriptional repressor of genes involved in anaerobic utilization of allantonin as a nitrogen source	AllZ	*grcA*	Stress-induced alternate pyruvate formate-lyase subunit; important in anaerobic maintenance of redox balance	Anaerobic metabolism	Cusa et al. (1999); Rintoul et al. (2002); Kramer et al. (2010)	*ftsA*.*ftsZ*.IGR; *ftsZ*; *bssR*
*bhsA*	Outer membrane stress protein; induced by H_2_O_2_ and increases cell resistance to H_2_O_2_ induced stress	BhsB	*ompC*	Outer membrane protein; was shown to facilitate and regulate the diffusion of H_2_O_2_ through the outer membrane in *Salmonella*	Cell response to oxidative stress	Pomposiello et al. (2001); Zheng et al. (2001); van der Heijden et al. (2016); Iwadate and Kato (2017)	
			*ytfK*	Stringent response activator; induced by paraquat, involved in H_2_O_2_ tolerance			
*fadA*	3-ketoacyl-CoA thiolase, involved in fatty acid degradation via β-oxidation and generation of acetyl-CoA	FadZ	*dctA*	C4 dicarboxylate/orotate:H^+^ symporter; importer of metabolites that can serve as substrates in the TCA cycle	TCA cycle	Kay and Kornberg (1969, 1971); Darlison et al. (1984); Buck et al. (1986)	*clpS*; *ompC*; *ompF*; *yhsB*
			*kgtP*	a-Ketoglutarate:H^+^ symporter			
			*sucA*	Component of the 2-oxoglutarate dehydrogenase multienzyme complex			
*kilR*	Killing protein; inhibits cell division by binding FtsZ	KilS	*yncL*	Inner membrane protein of unknown function	KilR targets FtsZ and YncL, both localize to the inner membrane	Overath and Raufuss (1967); Kay and Kornberg (1969, 1971); Lo et al. (1972)	

*^*a*^Only RIL-seq targets detected in at least one individual library in addition to the unified libraries were regarded in this analysis.*

In summary, using our methodology followed by experimental verification we reaffirmed that there is a rich repertoire of sRNAs encoded within various genomic elements and generated under different conditions. The use of our systematic approach has allowed us to identify putative sRNAs that would not have been considered otherwise, such as AllZ, KilS and BhsB. Each of them is involved in only a few interactions and a few hundred chimeric fragments, and they would not have been suspected as sRNAs by examining their individual features alone. Yet, the overall combination of their features has allowed their detection. Especially, their interactors had very few interactions with “other RNAs”, rejecting the interactors as sRNAs and supporting AllZ, KilS and BhsB as sRNAs. We believe that taking into account both the first- and second-layer features empowers our predictions. Hence, taking into account the information extracted directly from RIL-seq data while accounting for the RNA–RNA interaction network inferred from RIL-seq results is highly rewarding. Our approach may be generalized to other RNA-seq-based methodologies, where the results may imply a network structure or hierarchy of the genes. Combining features based on the direct sequencing results with features based on a higher-order structure of the data may prove beneficial to the inference of novel biological insights in other contexts.

## Data Availability Statement

The data used in this article can be found in https://www.ebi.ac.uk/arrayexpress/E-MTAB-9834 and in https://www.ebi.ac.uk/arrayexpress/E-MTAB-3910.

## Author Contributions

HM and YA initiated and supervised the study. LA contributed to the experimental investigation. AB contributed to the software development and computational analysis. YA, LA, AB, and HM contributed to the writing – original draft and review and editing. All authors contributed to the article and approved the submitted version.

## Conflict of Interest

The authors declare that the research was conducted in the absence of any commercial or financial relationships that could be construed as a potential conflict of interest.
