# Supplementary material for: Prediction of Novel Bacterial Small RNAs From RIL-Seq RNA–RNA Interaction Data
Source: Front Microbiol. 2021 May 21;12:635070. doi: 10.3389/fmicb.2021.635070 (PMC8175672; doi:10.3389/fmicb.2021.635070)
Supplement: Supplementary file 7 [file Data_Sheet_1.pdf]

Supplementary Material

Prediction of novel bacterial small RNAs from RIL-seq RNA-RNA interaction data

Amir Bar, Liron Argaman, Yael Altuvia and Hanah Margalit

TABLE OF CONTENTS

Computational Procedures

Supplementary Tables (excel files)
Supplementary Table 1. Feature values and sRNA scores for all RNAs in the data sets
Supplementary Table 2. Features considered for the predictive model
Supplementary Table 3. Oligonucleotides used in the northern blots carried out in the study
Supplementary Table 4. Weights of the logistic regression models
Supplementary Table 5. Target hubs and sRNA sponges
Supplementary Table 6. List of transcription start sites and RNase E cleavage sites in the vicinity of recently discovered and novel sRNAs

Supplementary Figures
Supplementary Figure 1. Clustering of correlated traits
Supplementary Figure 2. Distributions of the selected features for the data set of exponential phase
Supplementary Figure 3. Distributions of the selected features for the data set of exponential phase under iron limitation
Supplementary Figure 4. Detection of novel sRNAs in RIL-seq data of exponential phase
Supplementary Figure 5. Detection of novel sRNAs in RIL-seq data of exponential phase under iron limitation
Supplementary Figure 6. Association between the sRNA score and expression level
Supplementary Figure 7. Effect of model parameters on the predicted probabilities – exponential phase
Supplementary Figure 8. Effect of model parameters on the predicted probabilities – stationary phase
Supplementary Figure 9. Effect of model parameters on the predicted probabilities – exponential phase under iron limitation

Supplementary Figure 10. Contribution of the various features to the logistic regression predictions

Supplementary Figure 11. Expression patterns of RbsZ and MalH

Supplementary Figure 12. Coverage plots of RNA-seq in the loci of *ykgH* and *glpX*

Supplementary References

Computational Procedures

Feature selection

We examined 18 traits that could potentially contribute to distinguishing sRNAs from "other RNAs" (**Supplementary Table 2**). To identify traits that differ statistically significantly, we applied two-tailed Mann-Whitney U test to each feature and corrected the p-values for multiple hypotheses testing using Bonferroni correction. These tests were carried out for each data set separately and traits with corrected p-value > 0.05 in any of the data sets were discarded. Next, to remove redundant traits, we computed for each data set the absolute value of the Pearson correlation coefficient between every pair of traits, forming correlation matrices (**Supplementary Figure 1**). Clustering of each of these matrices (hierarchical clustering with an average link function) allowed us to identify groups of similar traits, implying their contribution to a predictive scheme might be redundant. In these cases, the computed values represented the same property, computed in different ways. For example, for the number of chimeric fragments a RNA was involved in, we considered three traits that were found to be correlated: the total number of chimeric fragments the RNA was involved in and the mean or median of its number of chimeric fragments across all its interactions. For each cluster of traits, we selected one representative trait to be included in further analysis (referred to as ’feature’ in the successive analyses). We attempted to select as representative the trait manifestation that was most intuitive. For example, for the number of chimeric fragments the RNA was involved in, we chose to represent the three correlated features by the first one, the total number of chimeric fragments the RNA was involved in, rather than the mean or median.

Selection of the model parameters

We run the logistic regression model 10,000 times (iterations), where in each iteration we use 2/3 of the data for training the model and 1/3 of the data for assessing the model predictions. To verify that our results are independent of the number of iterations and of the ratio between the training and test data set sizes, we ran the logistic regression analysis with different values of these parameters (10 values evenly spaced between 0.2 and 0.5 for the test set size, and 10 values evenly spaced on a log scale between 10^3 and $10^{4.5}$). As most of the RNAs in the data sets are of “other RNAs”, we expect most predictions to have probabilities close to zero. Thus, instead of measuring

the mean difference in sRNA probability for each RNA, we measured the sum over all RNAs in the data of absolute differences in probabilities $S_{i,j} = \sum_x |p_{X,i} - p_{X,j}|$ where $p_{X,i}$ is the sRNA probability of RNA 'X' with parameter combination *i* (number of iterations and ratio between the training and test set sizes). We examined several values for each parameter while fixing the other i.e., we used a baseline test size fraction of 1/3 and 10,000 model iterations (**Supplementary Figures 7A,B, 8A,B, 9A,B**). The total difference in prediction probabilities was around 0.1 when we used at least ~4500 iterations. Hence, we remained with our initial selection of 10,000 iterations. For assessing the effect of the training/test size ratio on the results, we computed the mean ROC and PR curve AUCs for a range of different ratios (**Supplementary Figure 7C,D, 8C,D, 9C,D**). The performances for the various values were within one standard deviation of the original values obtained for the ratio of 1:2. Hence, the results are independent of the model parameters.

Supplementary Tables (Excel files)

Supplementary Table 1. Feature values and sRNA scores for all RNAs in the data sets

The file includes a legend describing the various columns of the table, a sheet for the results for each data set, and a summary sheet. The table presents the feature values and computed sRNA score for each RNA in the different data sets. The summary sheet contains all the RNAs from the three data sets and the respective sRNA scores they obtained in the analyses of the various data sets, along with a note whether a sRNA was known, recently confirmed, confirmed here, or predicted but not yet tested.

Supplementary Table 2. Features considered for the predictive model

The file includes the description of all the traits considered in this study, and the results of the statistical tests for each of the data sets.

Supplementary Table 3. Oligonucleotides used in the northern blots carried out in the study

The file lists the sequences used as probes in the northern blots carried out in this study for each of the putative novel sRNAs.

Supplementary Table 4: Weights of the logistic regression models

The file contains the mean intercept and weights of the 10,000 logistic regression iterations for each data set.

Supplementary Table 5 - Target hubs and sRNA sponges

The file includes a legend describing the various columns of the table, and a sheet with two sub-tables: "target hubs" – RNAs that interact in at least one condition with at least four unique sRNAs Supplementary Table S2 in Melamed et al.(Melamed et al., 2016). sRNA sponges - RNAs defined as sponges (Adams et al., 2021; Denham, 2020) and are found in the RIL-seq data. sRNA scores and number of unique interactions from **Supplementary Table 1** is presented for each of the listed RNAs along with the number of unique interactions with sRNAs as described above.

Supplementary Table 6. List of transcription start sites and RNase E cleavage sites in the vicinity of recently discovered and novel sRNAs

The file includes genomic information about recently discovered sRNAs (listed in **Table 2A** in the main text) and the novel sRNAs studied here (listed in **Table 2B** in the main text). For each RNA, we report the genomic location of the RNA as previously published or as annotated in our data, adjacent transcriptions start sites, adjacent RNase E cleavage sites and predicted sRNA scores in each data set.

Supplementary Figures

A

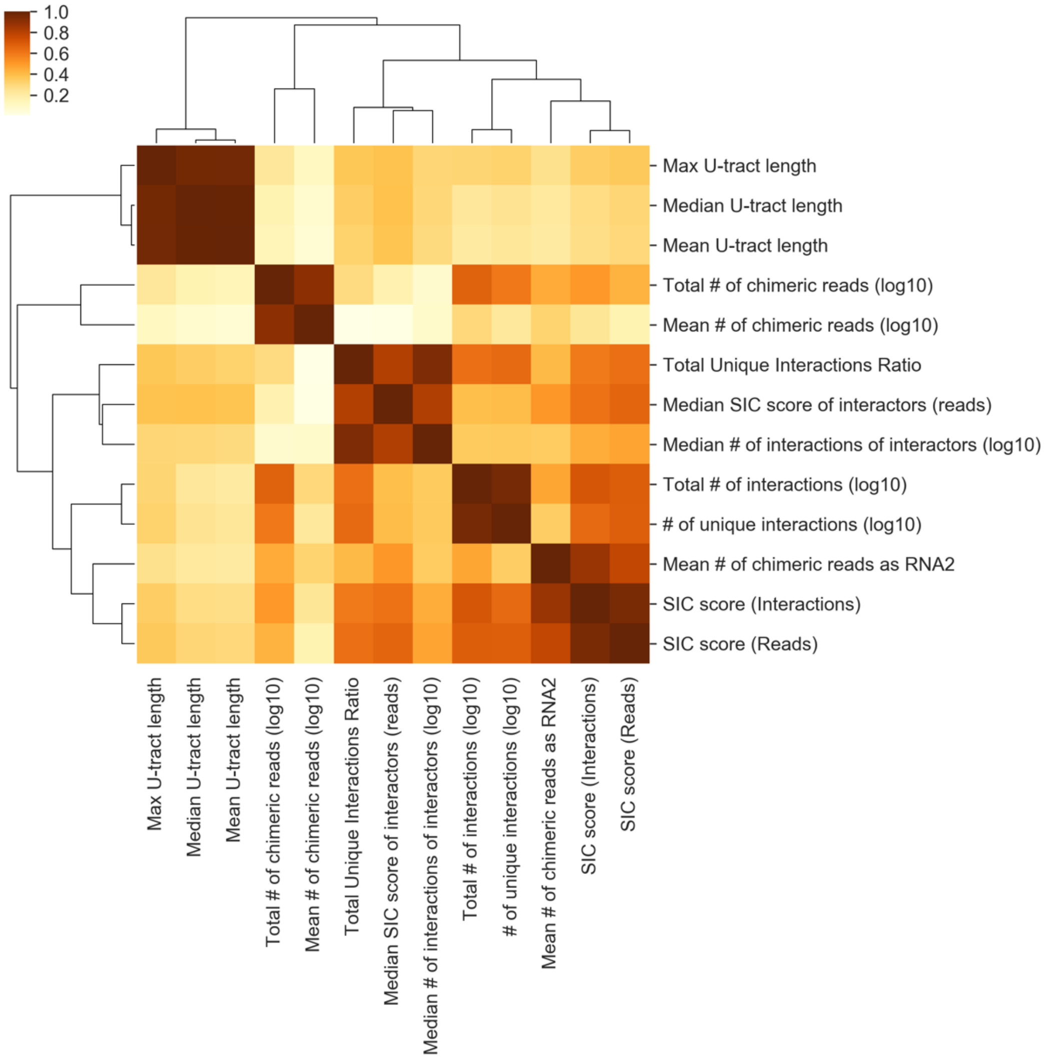

B

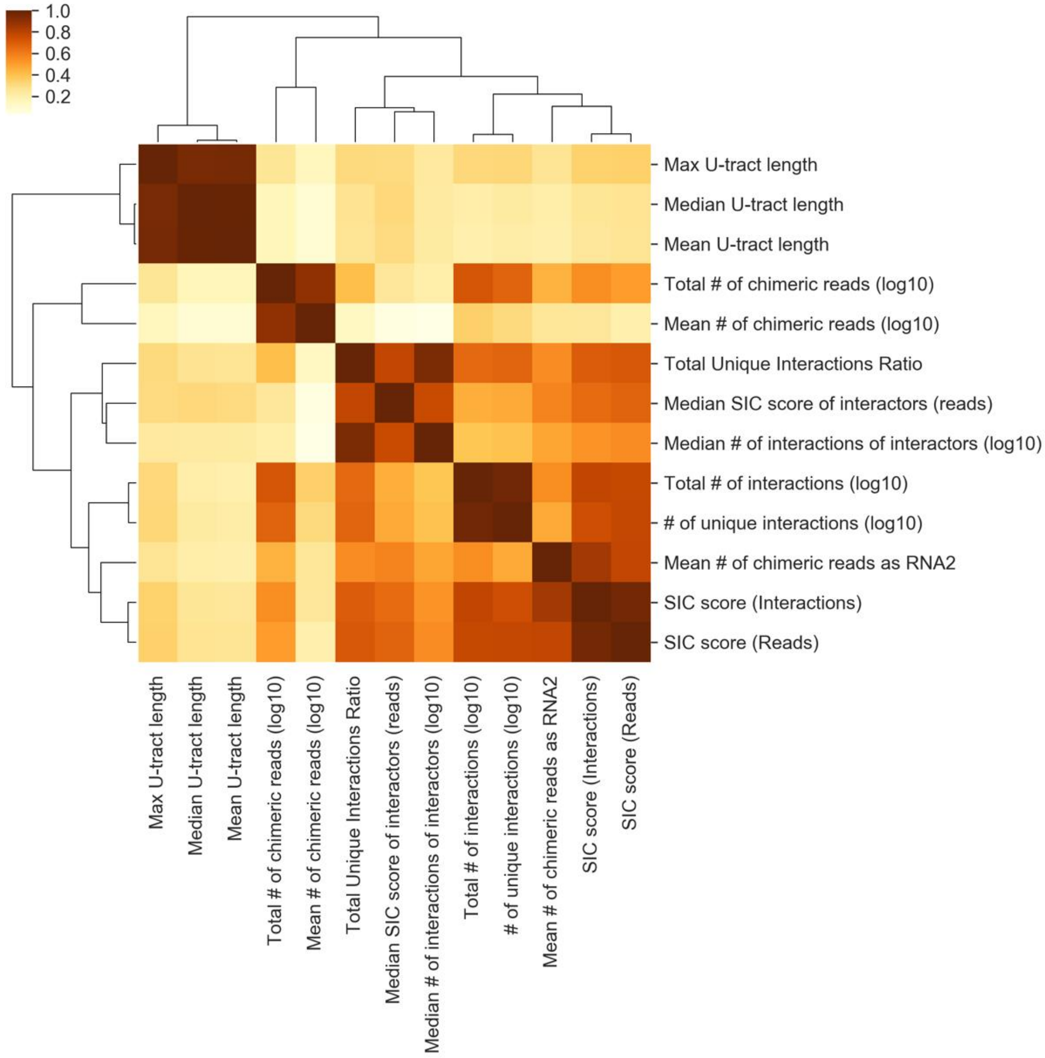
1.0
0.8
0.6
0.4
0.2
Max U-tract length
Median U-tract length
Mean U-tract length
Total # of chimeric reads (log10)
Mean # of chimeric reads (log10)
Total Unique Interactions Ratio
Median SIC score of interactors (reads)
Median # of interactions of interactors (log10)
Total # of interactions (log10)
# of unique interactions (log10)
Mean # of chimeric reads as RNA2
SIC score (Interactions)
SIC score (Reads)

C

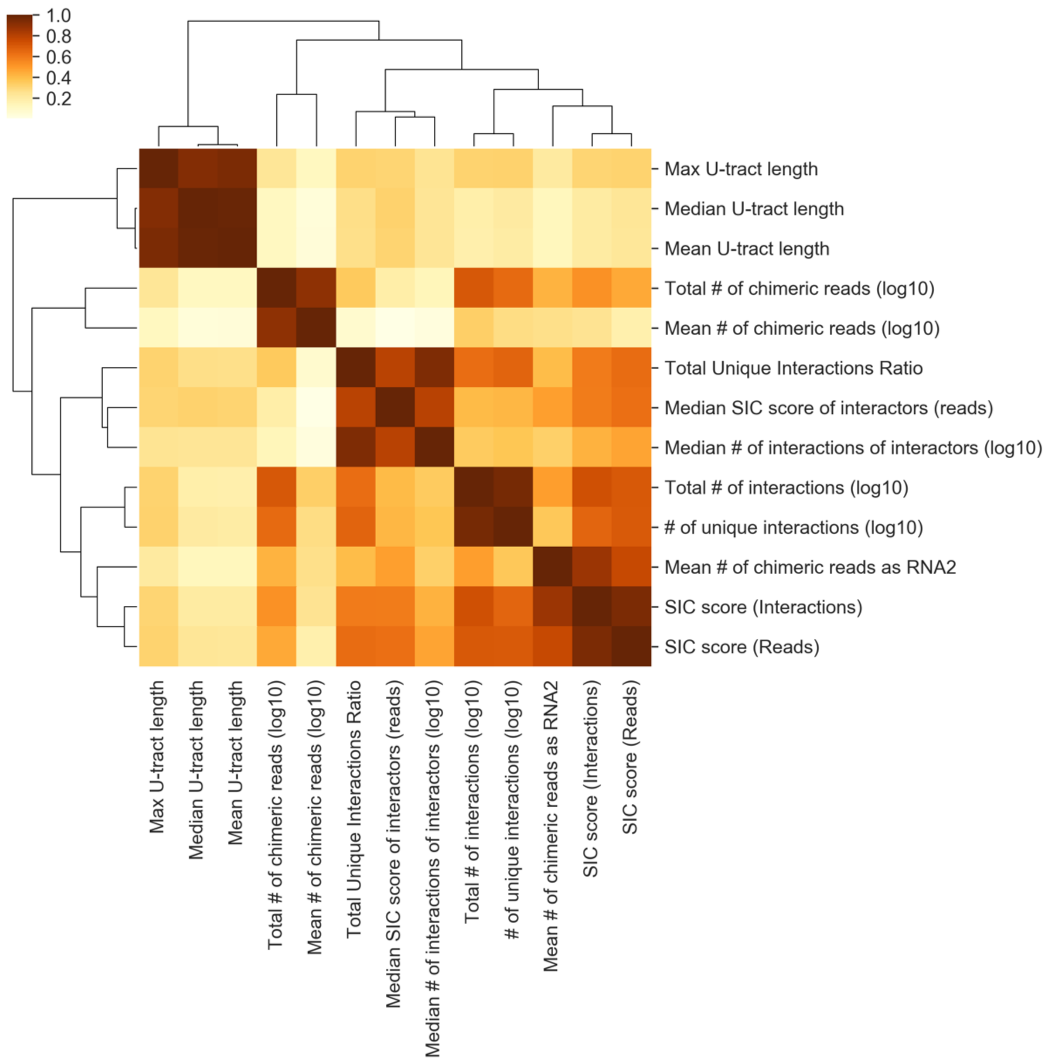

Supplementary Figure 1. Clustering of correlated traits
A heatmap presenting the similarity between statistically significantly differing traits for the data set of exponential phase **(A)**, stationary phase **(B)**, and exponential phase under iron limitation **(C)**. Each cell in the heatmap represents the absolute value of the correlation coefficient between the two features. The rows and columns were clustered with hierarchical clustering to group similar features.

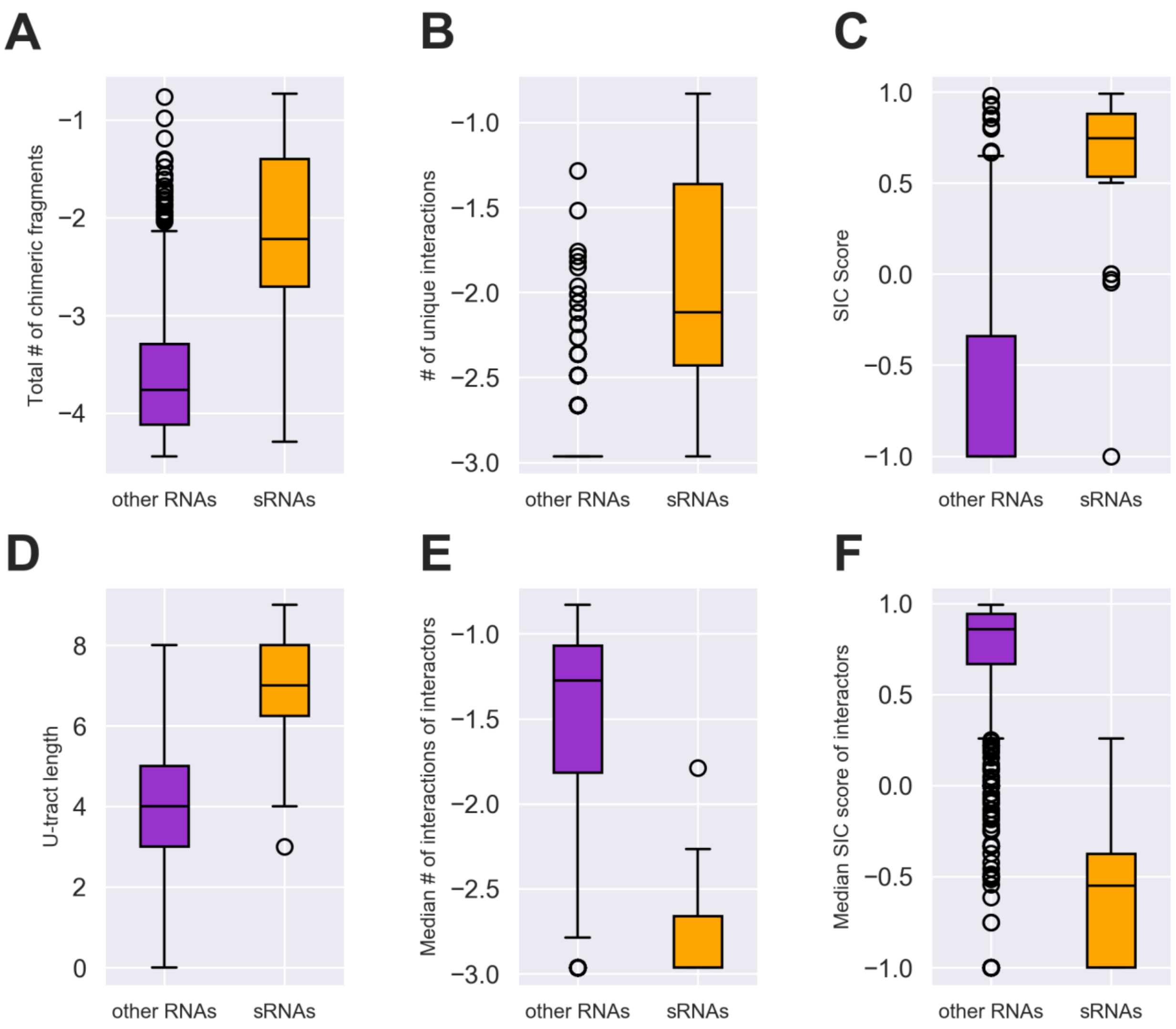

Supplementary Figure 2 (related to Figure 1). Distribution of the selected features for the data set of exponential phase

Each RNA was characterized by the following features: **(A)** Total number of the chimeric fragments the RNA is involved in. The value was normalized by the total number of chimeric fragments in the data set and then transformed to Log_{10} scale. **(B)** Number of unique interactions the RNA is involved in. The value was normalized by the total number of interactions and then transformed to Log_{10} scale. **(C)** SIC score, (SIC, for Second-in-Chimera), namely the percentage of chimeric fragments in which the RNA was second in the chimera, penalized by the number of unique interactions the RNA is involved in. **(D)** The length of the RNA U-tract. **(E)** The median number of interacting partners of the RNA interactors (normalized as in B and expressed by log_{10}). **(F)** The median SIC score of the RNA interactors. Described by boxplots are the distributions of each feature values in the group of known sRNAs (orange) and "other RNAs" (purple). The differences between the two distributions **(A-F)** were found to be statistically significant by two-tailed Mann-Whitney U test (p values between 10^{-17} and 10^{-10} after Bonferroni correction). The

distributions in panels **(A-F)** are based on the data of exponential phase RIL-seq experiment (**Supplementary Table 1**).

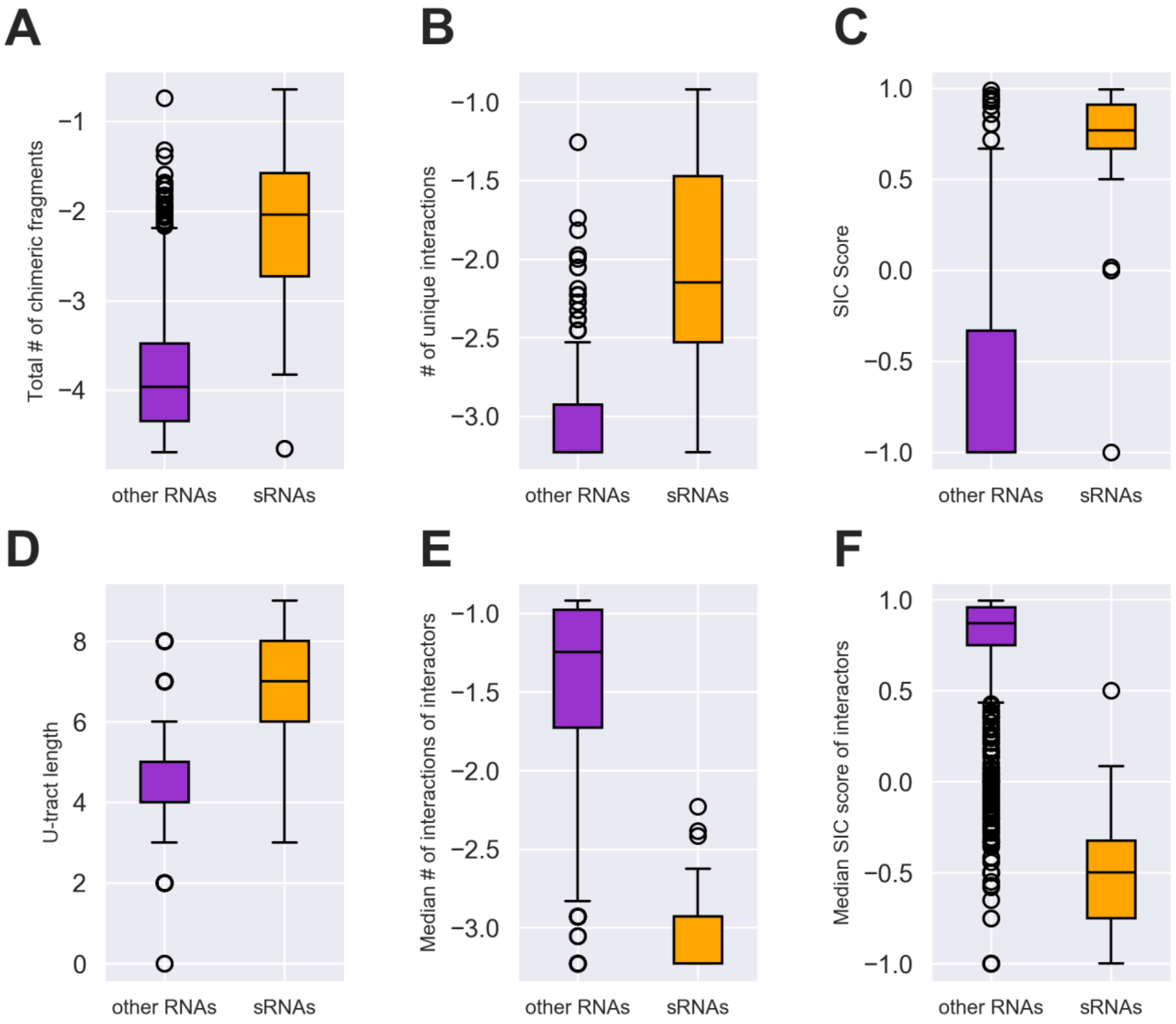

Supplementary Figure 3 (related to Figure 1). Distribution of the selected features for the data set of exponential phase under iron limitation

Figure legend as in **Supplementary Figure 4**. The differences between the two distributions **(A-F)** were found to be statistically significant by two-tailed Mann-Whitney U test (p values between 10^{-20} and 10^{-11} after Bonferroni correction). The distributions in panels **(A-F)** are based on the data of RIL-seq experiment in exponential phase under iron limitation (**Supplementary Table 1**).

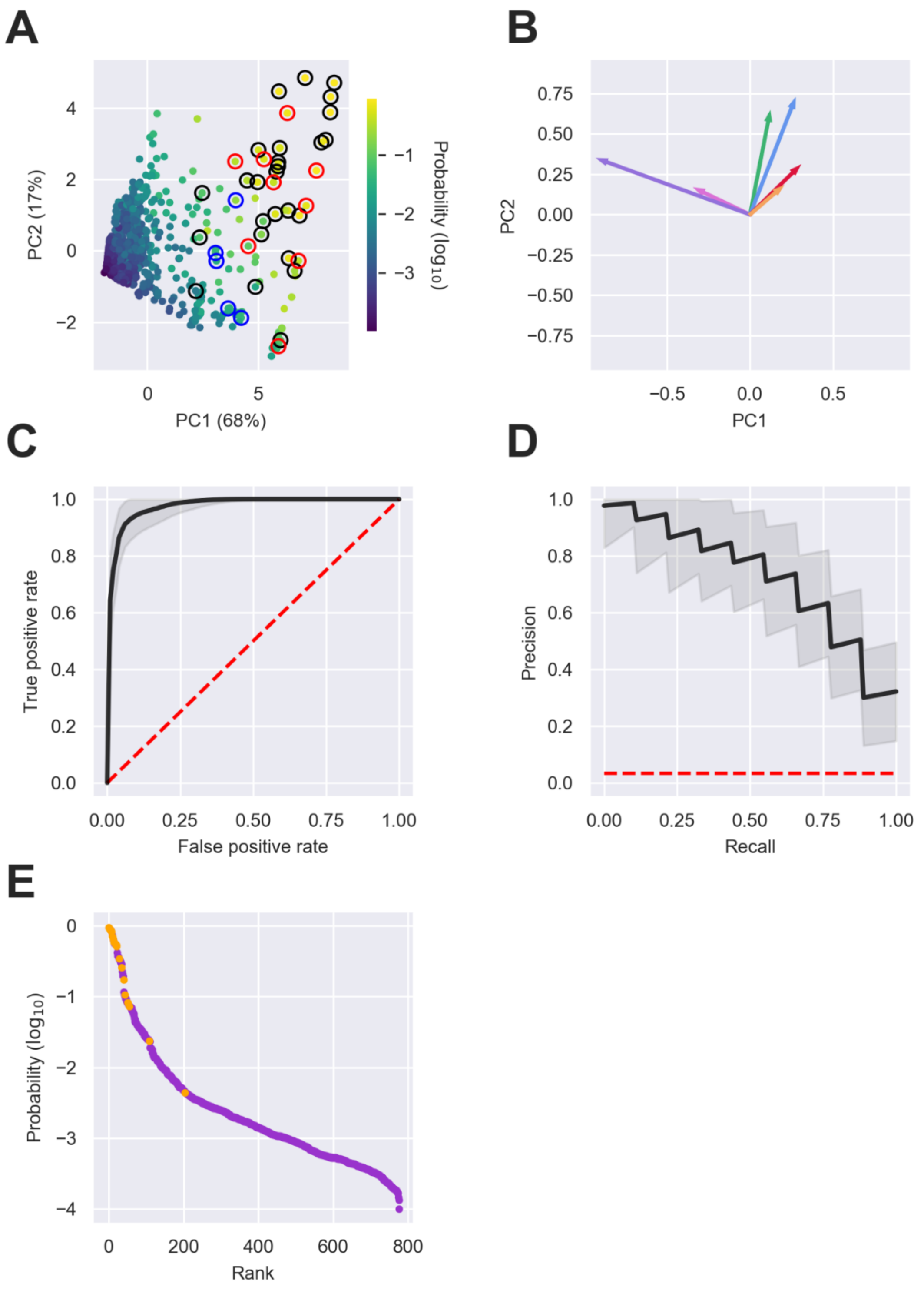

Supplementary Figure 4. Detection of novel sRNAs in RIL-seq data of exponential phase
(A) Principal Component Analysis (PCA) of RNAs characterized by the six features. The RNAs (dots) are plotted in two dimensions, using their projections onto the first two principal components. Each RNA in the data is colored by its sRNA probability, as assigned by the logistic regression analysis. Colored circles surrounding the dots represent: a well-established sRNA

marked in **Supplementary Table 1** by 1 (black), a recently discovered sRNA listed in **Table 2A** (red) or a newly discovered sRNA listed in **Table 2B** (blue). **(B)** Contribution of the features to PC1 and PC2. The vectors represent the coefficients of the features in each PC: Total number of chimeric fragments (green), number of unique interactions (blue), SIC score (red), U-tract length (orange), median number of interactions of interactors (pink), median SIC score of interactors (purple). **(C-D)** Receiver operating characteristic (ROC) curve **(C)** and Precision-Recall (PR) curve **(D)** showing the high predictive power of the logistic regression model. Shown in black are the curves obtained from the mean probabilities of 10,000 iterations of the logistic regression, and the curves of individual iterations in the range of one standard deviation around the curve of mean probabilities. The curves are compared to the expected curve of a random classifier (red dashed line). The area under curve (AUC) of the ROC curve is 0.98±0.02. **(E)** known sRNAs and "other RNAs" (colored orange and purple, respectively) were ranked by their computed sRNA scores. Highly ranked RNAs, yet unknown as sRNAs, are predicted as putative novel sRNAs. Results are for the data set of exponential phase RIL-seq experiment.

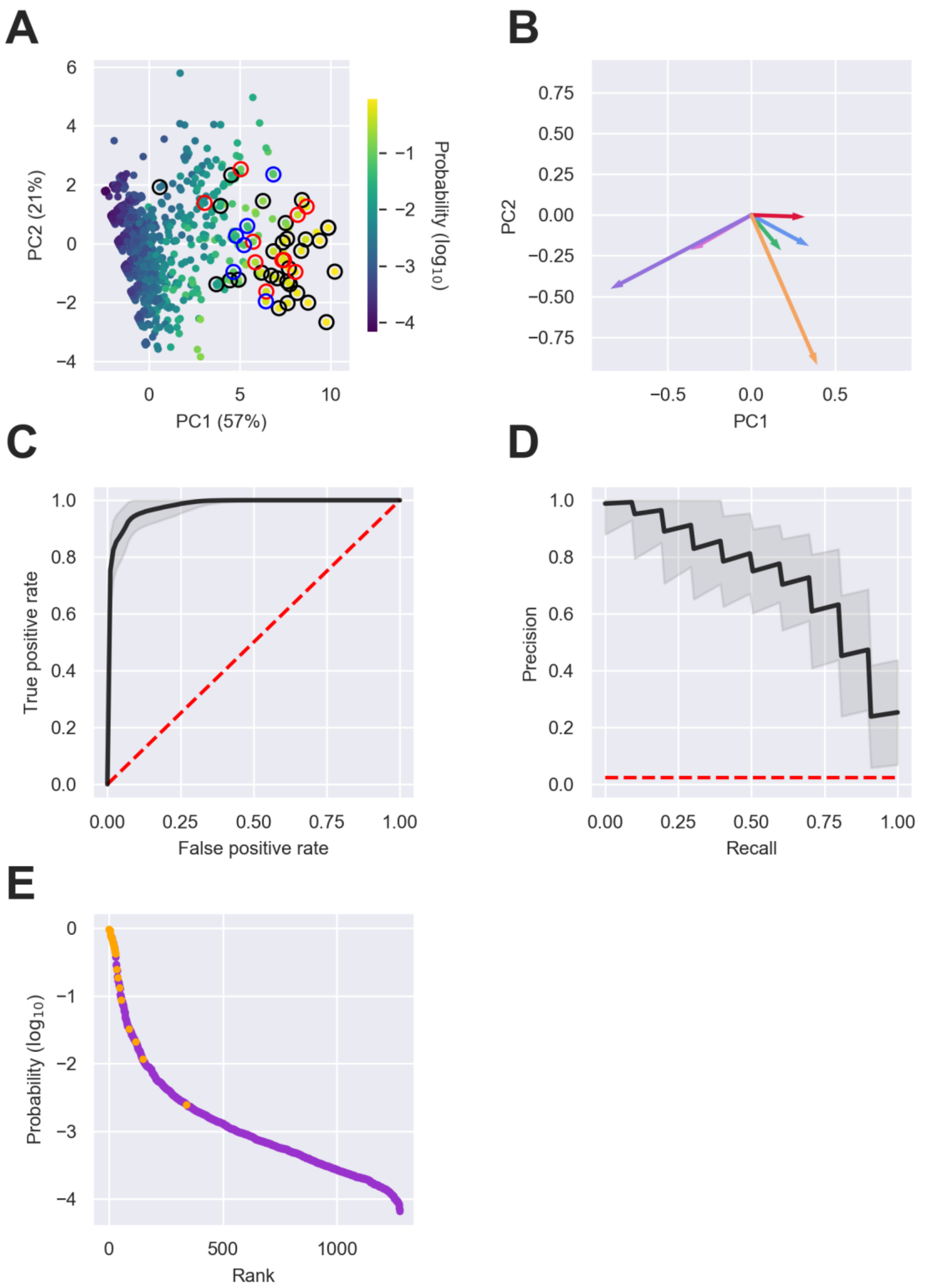

Supplementary Figure 5. Detection of novel sRNAs in RIL-seq data of exponential phase under iron limitation

Figure legend as in **Supplementary Figure 4**. The area under curve (AUC) of the ROC curve **(C)** is 0.98±0.01. Results are for the data set of RIL-seq experiment in exponential phase under iron limitation.

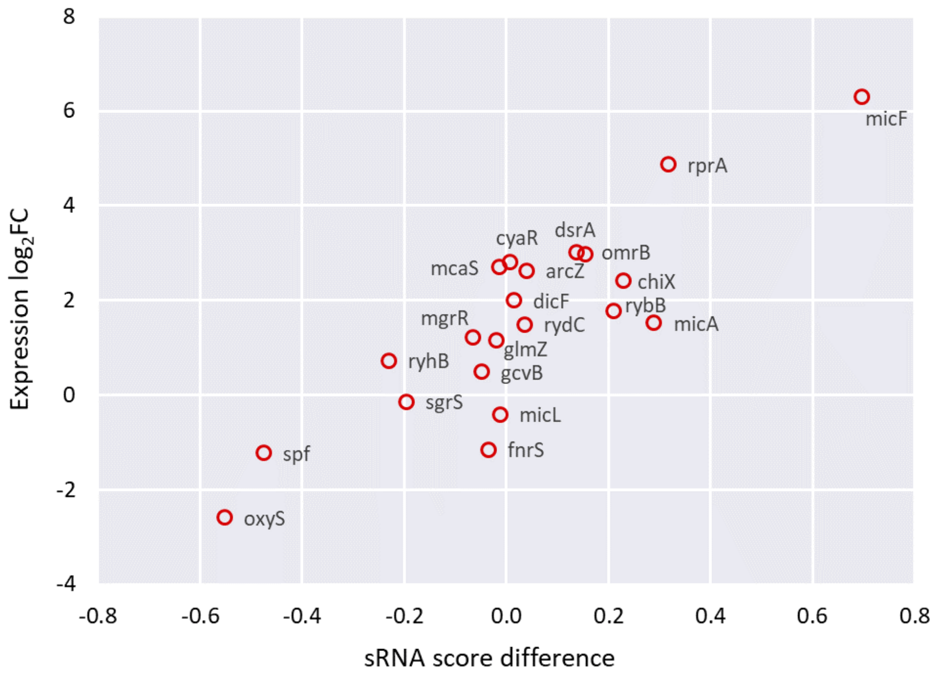

Supplementary Figure 6. Association between the sRNA score and expression level
Shown are values for 21 RNAs annotated as sRNAs in Melamed et al, (2016) which were involved in at least one interaction in both RIL-seq data sets of exponential phase and stationary phase. The sRNA score difference is based on **Supplementary Table 1** and was computed by subtracting the sRNA score computed for exponential phase data from the sRNA score computed for stationary phase data. The difference in the RNA expression level (Expression $\log_2$FC) between stationary phase and exponential phase was computed by DESeq2 (Love et al., 2014) applied to RNA-seq expression data from Melamed et al, (2016) (ArrayExpress E-MTAB-3910). Three additional sRNAs from Melamed et al, (2016) RyjA, SdsR, OmrA did not have interactions in the exponential phase data but did have interactions in the stationary phase data. Consistently, they had high $\log_2$FC values of 6.6, 7.1 and 4.2, respectively. SdsR and OmrA had high sRNA scores (0.4 and 0.6, respectively) while RyjA, which had only 2 interactions, had a low score of 0.002.

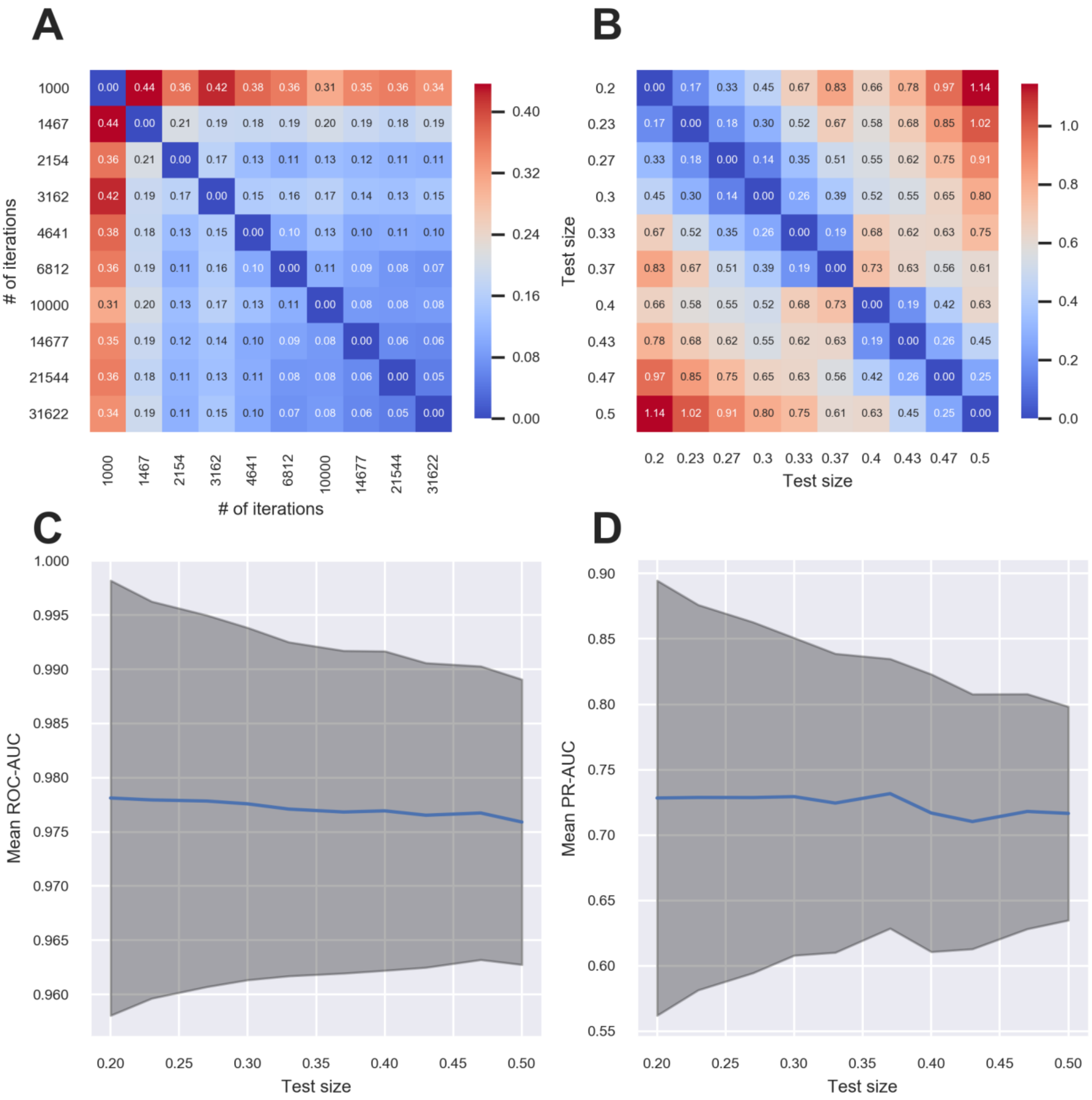

Supplementary Figure 7. Effect of model parameters on prediction accuracy - exponential phase data

(A-B) A heatmap representing the absolute total change in prediction probabilities when running the model with a different number of iterations **(A)** or a different fraction of test size **(B)**. Each cell contains the sum of absolute differences between the probabilities of each RNA in the data set. **(C-D)** The test size effect on the model's mean AUC (blue line) of the ROC **(C)** and PR **(D)** curves with one standard deviation (dark gray area).

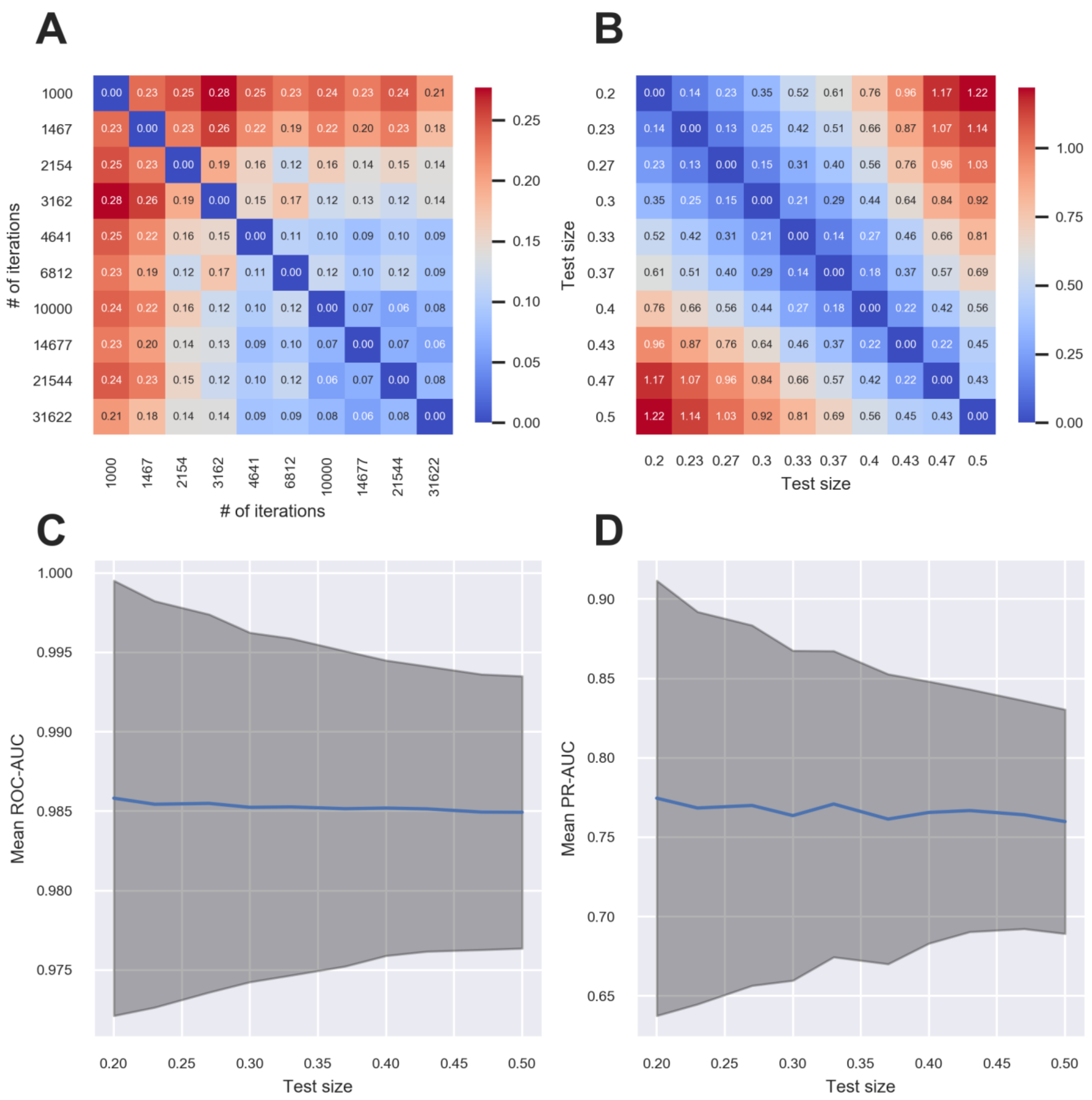

Supplementary Figure 8. Effect of model parameters on prediction accuracy - stationary phase data

Figure legend as in **Supplementary Figure 7**. Results are for the data set of RIL-seq experiment in stationary phase.

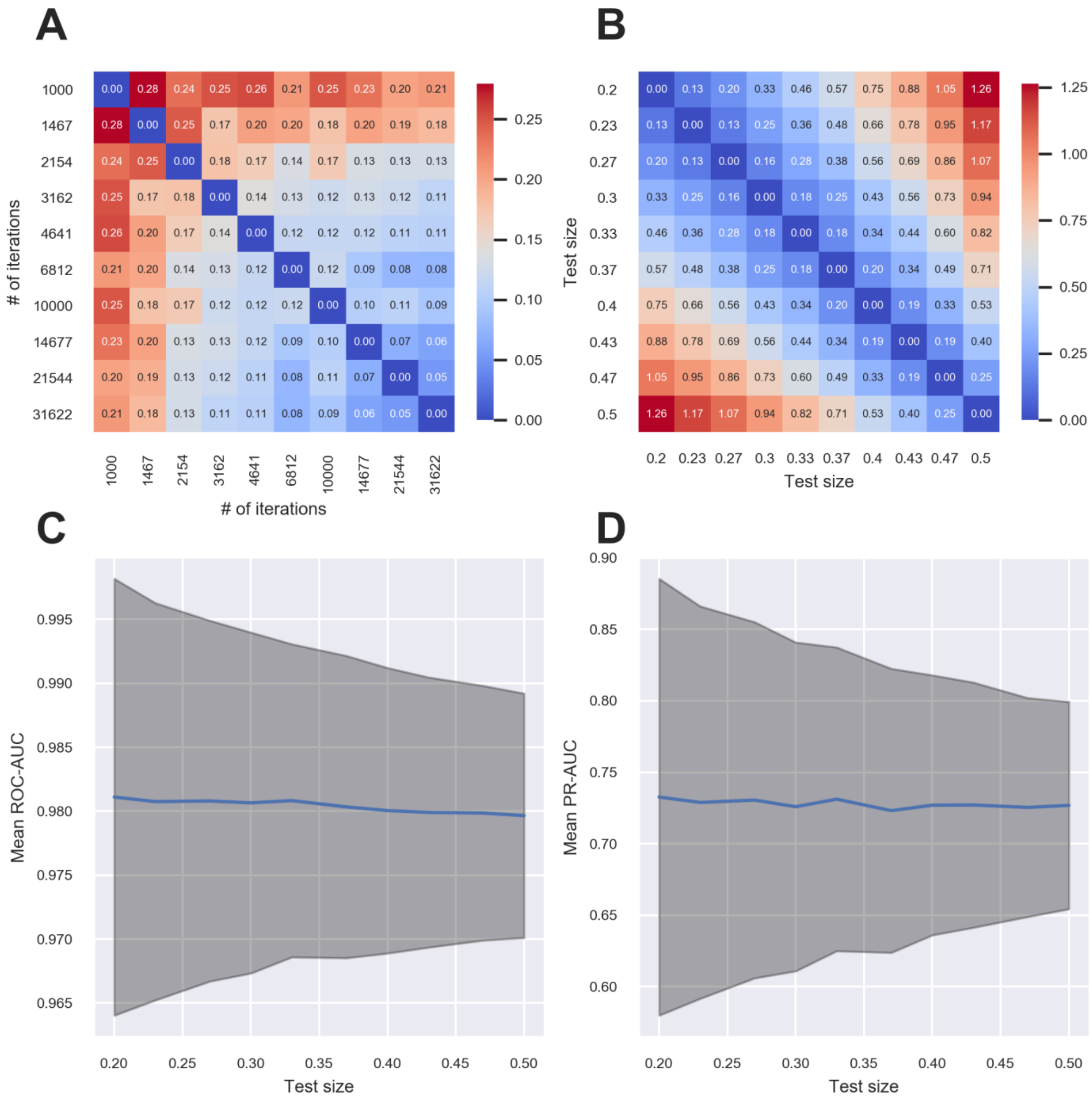

Supplementary Figure 9. Effect of model parameters on prediction accuracy - exponential phase under iron limitation data

Figure legend as in **Supplementary Figure 7**. Results are for the data set of RIL-seq experiment in exponential phase under iron limitation.

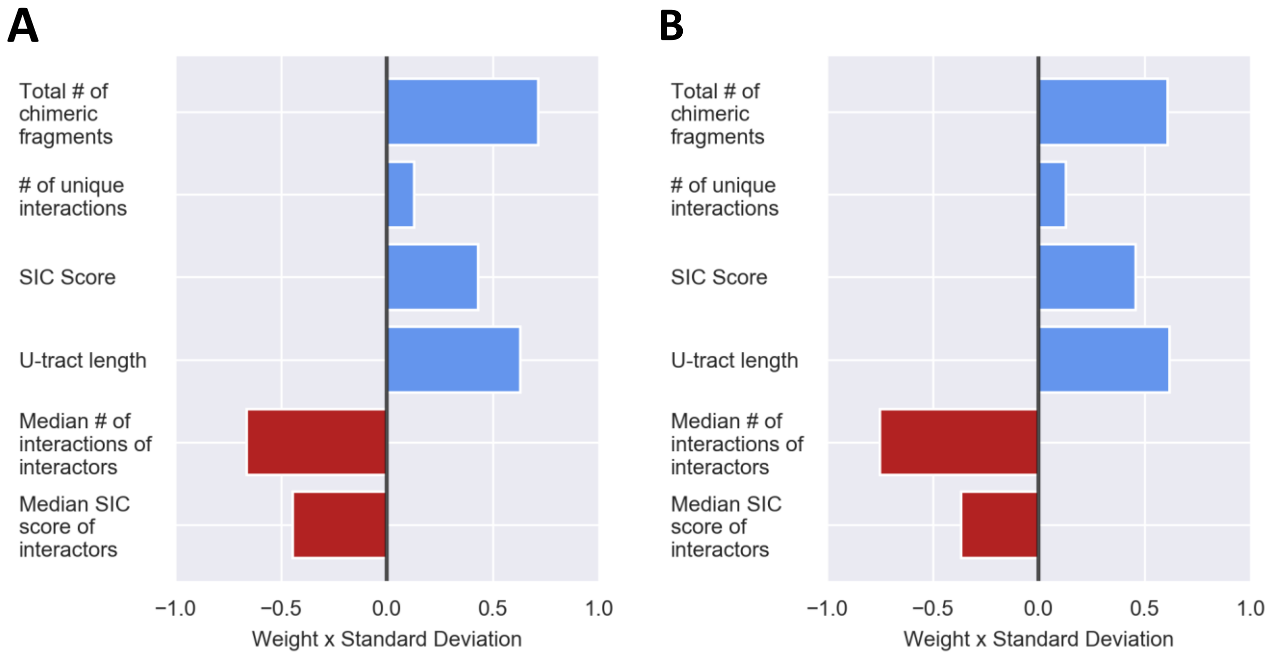

Figure 10. Contribution of the various features to the logistic regression predictions
Presented are the logistic regression weights after z-score transformation of the feature values (see Materials and Methods in the main text). The presented weights, which are the original weights (**Supplementary Table 4**) multiplied by the standard deviation of the feature value, are comparable. The weight value represents its contribution to the probability the logistic regression model provides, and the sign signifies the direction in which the weight affects this probability (i.e., positive values increase the sRNA probability and negative values reduce the sRNA probability). The results are based on the data set of RIL-seq experiments in exponential phase **(A)** and exponential phase under iron limitation **(B)**.

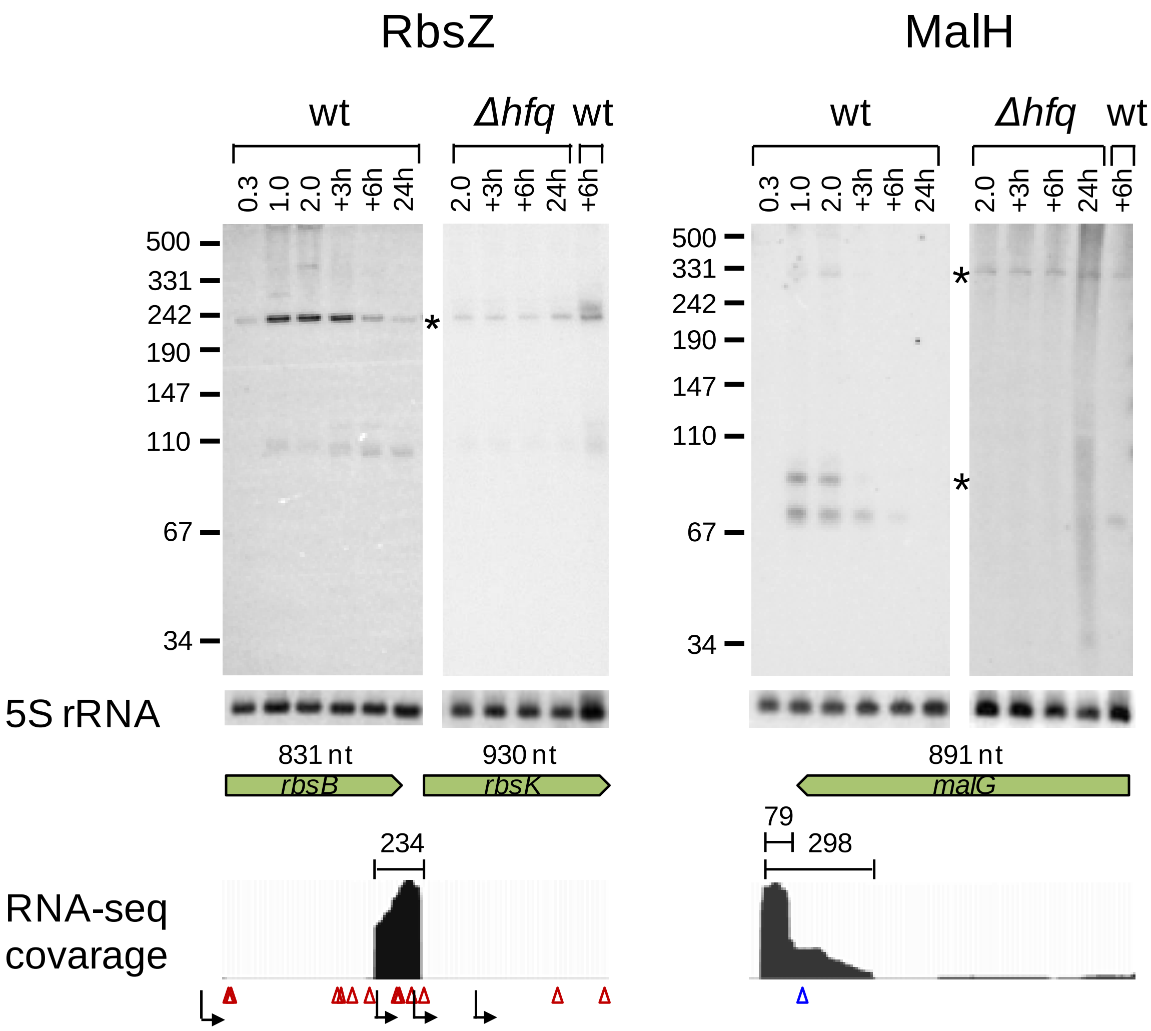

Supplementary Figure 11. Expression patterns of RbsZ and MalH

Total RNA was extracted from wt *E. coli* and *Δhfq* cultures throughout growth. Samples of the wt culture were taken at an OD_{600} of 0.3, 1.0 and 2.0, 3 hr and 6 hr after the culture reached an OD_{600} of 2.0 (+3h and +6h, respectively) and after 24 hr of growth (24h). Samples of the *Δhfq* were taken at an OD_{600} of 2.0, 3 hr and 6 hr after the culture reached an OD_{600} of 2.0 and after 24 hr of growth. 30 µg Total RNA were subjected to northern analysis using specific probes. The sample of wt +6 was used as a reference sample in the *Δhfq* blots. 5S rRNA was probed as a loading control. For each sRNA, a coverage plot of RNA-seq library made of total RNA from a stationary phase (6 hr growth) culture is shown. The green arrows indicate the coding sequence region (CDS) and gene orientation, with the CDS size above the arrow in nucleotides (nt). The approximated size of each sRNA is indicated above the read coverage plot (nt). Transcription start sites (bent black arrows) and RNase E cleavage sites (red triangles) based on data of (Ju et al., 2019; Thomason et al., 2015) and (Clarke et al., 2014), respectively, are shown below the read coverage plots along the transcript. The site in which two adjacent 5' ends of MalH were mapped by Iosub et al. (Iosub et al., 2020) is represented by a blue triangle. Transcription start and cleavage sites in the vicinity of the suspected sRNA are recorded also in **Supplementary Table 6**.

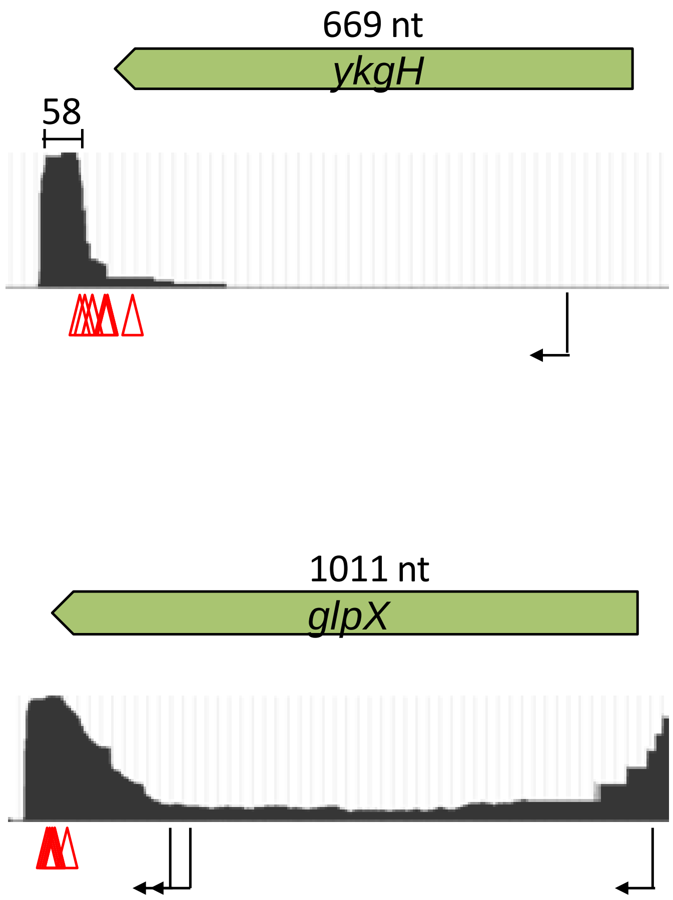

Supplementary Figure 12. Coverage plots of RNA-seq in the loci of *ykgH* and *glpX*
RNA-seq library was made of total RNA from a stationary phase (6 hr growth) culture. The green arrows indicate the coding sequence region (CDS) and gene orientation, with the CDS size above the arrow in nucleotides (nt). The approximated size of the putative sRNA encoded at the *ykgH* 3' UTR is indicated above the *ykgH* read coverage plot (nt). Transcription start sites, based on data of Thomason et al. (Thomason et al., 2015) and Ju et al. (Ju et al., 2019), and RNase E cleavage sites, based on data of Clarke et al. (Clarke et al., 2014), are shown below the read coverage plots along the transcript by bent black arrows and red triangles, respectively. Transcription start and cleavage sites in the vicinity of the suspected sRNA are recorded also in **Supplementary Table 6**.

Supplementary References

Adams, P.P., Baniulyte, G., Esnault, C., Chegireddy, K., Singh, N., Monge, M., Dale, R.K., Storz, G., and Wade, J.T. (2021). Regulatory roles of Escherichia coli 5' UTR and ORF-internal RNAs detected by 3' end mapping. eLife *10*.

Clarke, J.E., Kime, L., Romero A., D., and McDowall, K.J. (2014). Direct entry by RNase E is a major pathway for the degradation and processing of RNA in *Escherichia coli*. Nucleic Acids Res *42*, 11733-11751.

Denham, E.L. (2020). The Sponge RNAs of bacteria - How to find them and their role in regulating the post-transcriptional network. Biochim Biophys Acta Gene Regul Mech *1863*, 194565.

Iosub, I.A., van Nues, R.W., McKellar, S.W., Nieken, K.J., Marchioretto, M., Sy, B., Tree, J.J., Viero, G., and Granneman, S. (2020). Hfq CLASH uncovers sRNA-target interaction networks linked to nutrient availability adaptation. eLife *9*.

Ju, X., Li, D., and Liu, S. (2019). Full-length RNA profiling reveals pervasive bidirectional transcription terminators in bacteria. Nature microbiology *4*, 1907-1918.

Love, M.I., Huber, W., and Anders, S. (2014). Moderated estimation of fold change and dispersion for RNA-seq data with DESeq2. Genome Biol *15*, 550.

Melamed, S., Peer, A., Faigenbaum-Romm, R., Gatt, Y.E., Reiss, N., Bar, A., Altuvia, Y., Argaman, L., and Margalit, H. (2016). Global Mapping of Small RNA-Target Interactions in Bacteria. Mol Cell *63*, 884-897.

Thomason, M.K., Bischler, T., Eisenbart, S.K., Förstner, K.U., Zhang, A., Herbig, A., Nieselt, K., Sharma, C.M., and Storz, G. (2015). Global transcriptional start site mapping using differential RNA sequencing reveals novel antisense RNAs in Escherichia coli. J Bacteriol *197*, 18-28.
